# A whole-body imaging technique for tumor-specific diagnostics and screening of B7H3-targeted therapies

**DOI:** 10.1172/JCI186388

**Published:** 2025-01-23

**Authors:** Lei Xia, Yan Wu, Yanan Ren, Zhen Wang, Nina Zhou, Wenyuan Zhou, Lixin Zhou, Ling Jia, Chengxue He, Xiangxi Meng, Hua Zhu, Zhi Yang

**Affiliations:** 1 Key laboratory of Carcinogenesis and Translational Research (Ministry of Education), Beijing Key Laboratory of Research, Investigation and Evaluation of Radiopharmaceuticals, NMPA Key Laboratory for Research and Evaluation of Radiopharmaceuticals (National Medical Products Administration), Department of Nuclear Medicine, Peking University Cancer Hospital and Institute, Beijing, China and; 2 Key Laboratory of Carcinogenesis and Translational Research (Ministry of Education, Beijing), Department of Pathology, Peking University Cancer Hospital and Institute, Beijing, China.; 3 Department of Nuclear Medicine, Affiliated Hospital of Zunyi Medical University, Zunyi, Guizhou, China.; 4 Key Laboratory of Carcinogenesis and Translational Research (Ministry of Education, Beijing), Department of Hepato-Pancreato-Biliary Surgery, Sarcoma Center, Peking University Cancer Hospital and Institute, Beijing, China.

**Keywords:** Clinical trials, Development, Oncology, Cancer, Molecular diagnosis

## Abstract

**BACKGROUND:**

B7H3, also known as CD276, is notably overexpressed in various malignant tumor cells in humans, with extremely high expression rates. The development of a radiotracer that targets B7H3 may provide a universal tumor-specific imaging agent and allow the noninvasive assessment of the whole-body distribution of B7H3-expressing lesions.

**METHODS:**

We enhanced and optimized the structure of an affibody (ABY) that targets B7H3 to create the radiolabeled radiotracer [^68^Ga]Ga-B7H3-BCH, and then, we conducted both foundational experiments and clinical translational studies.

**RESULTS:**

[^68^Ga]Ga-B7H3-BCH exhibited high affinity (equilibrium dissociation constant [K_D_] = 4.5 nM), and it was taken up in large amounts by B7H3-transfected cells (A549^CD276^ and H1975^CD276^ cells); these phenomena were inhibited by unlabeled precursors. Moreover, PET imaging of multiple xenograft models revealed extensive [^68^Ga]Ga-B7H3-BCH uptake by tumors. In a clinical study including 20 patients with malignant tumors, the [^68^Ga]Ga-B7H3-BCH signal aggregated in both primary and metastatic lesions, surpassing fluorine-18 fluorodeoxyglucose (^18^F-FDG) in overall diagnostic efficacy for tumors (85.0% vs. 81.7%), including differentiated hepatocellular and metastatic gastric cancers. A strong correlation between B7H3 expression and [^68^Ga]Ga-B7H3-BCH uptake in tumors was observed, and B7H3 expression was detected with 84.38% sensitivity and 100% specificity when a maximum standardized uptake value (SUVmax) of 3.85 was set as the cutoff value. Additionally, B7H3-specific PET imaging is expected to predict B7H3 expression levels in tumor cells, intratumoral stroma, and peritumoral tissues.

**CONCLUSION:**

In summary, [^68^Ga]Ga-B7H3-BCH has potential for the noninvasive identification of B7H3 expression in systemic lesions in patients with malignant tumors. This agent has prospects for improving pretreatment evaluation, predicting therapeutic responses, and monitoring resistance to therapy in patients with malignancies.

**TRIAL REGISTRATION:**

ClinicalTrials.gov NCT06454955.

**FUNDING:**

This research was financially supported by the Natural Science Foundation of Beijing Municipality (no. 7242266), the National Natural Science Foundation of China (no. 82202201), and the Young Elite Scientists Sponsorship Program by China Association for Science and Technology (CAST) (no. YESS20220230).

## Abstract 2

An affibody radiotracer targeting B7H3 for PET imaging of various malignant tumors and for non-invasive screening of B7H3 expression in tumor.

## Graphical abstract

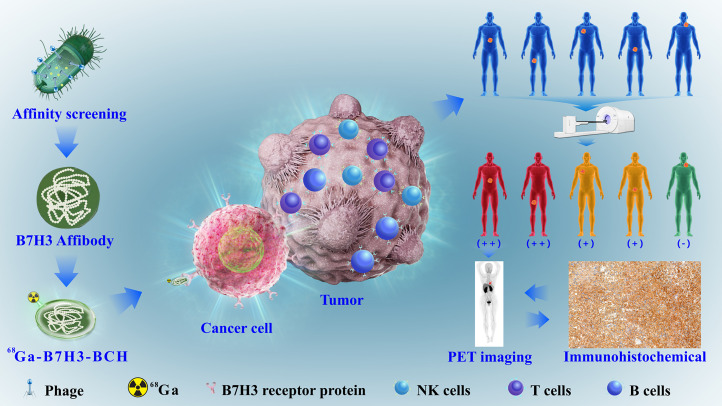

## Introduction

B7H3, which is sometimes called CD276, is a transmembrane glycoprotein B7 family member and a T cell regulator. B7H3 is notably and selectively overexpressed in different subtypes of human malignant tumor cells compared with normal tissues and benign lesions (1, 2). B7H3 is a cell-surface receptor protein that is closely associated with tumor resistance, metastasis, and immune modulation (3–5). Additionally, B7H3 is overexpressed on endothelial cells of the tumor vasculature, while it is expressed at low levels or not expressed in normal tissues (6–8). These characteristics make B7H3 an ideal candidate target for therapeutic agents that aim to ablate tumor cells and the tumor vasculature with high specificity. According to recent studies, B7H3-targeted antibody-drug conjugates (ADCs) demonstrate considerable potential for the treatment of various types of tumors. In fact, multiple pharmaceutical companies have begun to develop strategies that target this antigen (9–11). Academically, the presence and abundance of a target are still considered critical factors that determine the therapeutic efficacy of targeted treatment approaches (12). However, the methods for detecting B7H3 expression in tumors are still limited to invasive histopathological examinations. Because of the heterogeneity of tumors, determining the expression of therapeutic targets around and within metastatic lesions is challenging; this complicates pretreatment assessments of patient responses to treatment (13). With the development of targeted radiotracers, nuclear medicine techniques allow the high-specificity diagnosis of systemic B7H3 expression. Such methods qualitatively and quantitatively reveal receptor expression in primary and systemic metastatic lesions, thus facilitating a precise diagnosis and predicting the efficacy and prognosis of targeted therapies (14–16).

Currently, radionuclide therapeutic agents that target B7H3 have reached the forefront of clinical research. Kramer et al. (17) designed a targeted radionuclide therapeutic radiotracer by labeling a monoclonal antibody with ^131^I. The findings revealed that the radionuclide probe was safe, and survival was increased compared with the historical data among patients treated for neuroblastoma. Additionally, Burvenich et al. (18) constructed a targeted PET imaging probe using an anti-B7H3 antibody that was labeled with a radionuclide with a long half-life; this probe achieved favorable imaging results in animal models. However, the widespread clinical application of antibody-based radiotracers is limited by several challenges, including prohibitive costs and the potential of these radiotracers to elicit immune responses, especially under conditions of repeated administration (19). Previous research has shown that smaller protein fragments can facilitate efficient, locus-specific binding, and such fragments are increasingly replacing antibodies in tumor diagnosis research (20, 21). Affibodies (ABYs), which are promising binding ligands for designing molecular imaging tools, are small, 58–amino acid proteins with a molecular weight of approximately 7 kDa (22, 23). Compared with antibodies, ABYs demonstrate faster nonspecific clearance, greater biocompatibility, and better stability both in vivo and in vitro, making them better suited for widespread production and site-specific binding (24). A recent report highlighted the use of a highly specific ABY named AC12 to target B7H3, and AC12 has robust affinity, favorable biocompatibility, and optimal pharmacokinetic properties (25). A series of foundational studies were previously reported (26, 27). Oroujeni et al. (28) used this affinity ligand to construct a ^99m^Tc-labeled imaging probe, which achieved favorable single-photon emission computed tomography (SPECT) imaging results in a mouse tumor model. It is believed that such an ABY would be well suited for adaptation for use in PET molecular imaging.

In this study, we aimed to develop a radiotracer, namely, [^68^Ga]Ga-B7H3-BCH, which specifically targets B7H3 for use in PET imaging of various tumor types and to advance this radiotracer into clinical translation studies. This radiotracer was designed to allow the specific, noninvasive evaluation of B7H3 expression in all bodily lesions, and it is expected to overcome some limitations of nonspecific false-negative results that are inherent to fluorine-18 fluorodeoxyglucose (^18^F-FDG) imaging. Furthermore, we explored the potential effect of tumor B7H3 distribution on the uptake of this targeted radiotracer. This advanced capability is expected to enable the exhaustive exploration of the biological interactions among the radiotracer, the B7H3 protein, and cellular components.

## Results

### B7H3-targeting ABY improvement, synthesis, and quality control.

**Figure 1:**
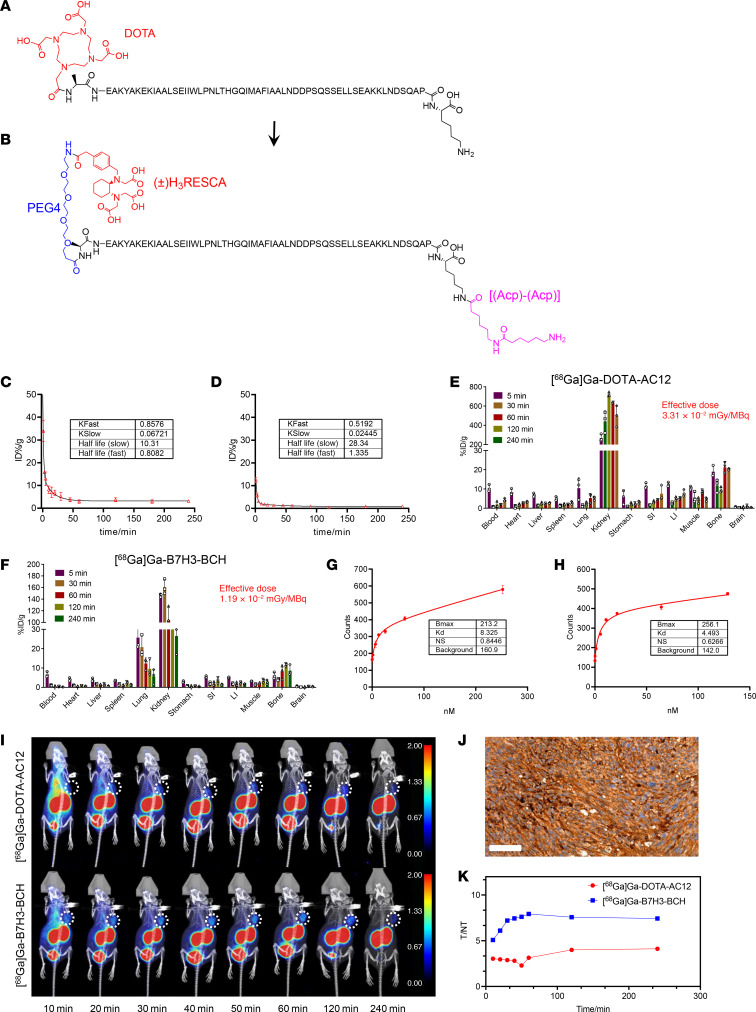
ABY improvement, synthesis, and quality control. (**A**) Chemical structure of DOTA-AC12. (**B**) Chemical structure of the improved RESCA-B7H3-BCH. (**C**) Pharmacokinetic parameters of ^68^Ga-DOTA-AC12 in vivo. (**D**) Pharmacokinetic parameters of [^68^Ga]Ga-B7H3-BCH in vivo. (**E** and **F**) Distribution and radiation dose estimation of ^68^Ga-DOTA-AC12 and [^68^Ga]Ga-B7H3-BCH in mice. (**G**) The binding affinity assays of ^68^Ga-DOTA-AC12. (**H**) The binding affinity assays of [^68^Ga]Ga-B7H3-BCH. (**I**) Head-to-head dynamic PET/CT imaging using ^68^Ga-DOTA-AC12 and [^68^Ga]Ga-B7H3-BCH in an H1975^CD276^ xenograft model. (**J**) B7H3 IHC of H1975^CD276^ tumor slice (scale bar: 100 μm). (**K**) Dynamic changes in the T/NT ratio by analyzing the SUVmax of ^68^Ga-DOTA-AC12 and [^68^Ga]Ga-B7H3-BCH PET/CT images.

Our initial efforts focused on replicating the synthesis of the AC12 structure and conjugating it with the bifunctional chelator DOTA to create a molecular probe (Figure 1A); additionally, the resulting probe was subjected to rigorous quality control (Supplemental Figure 1; supplemental material available online with this article; https://doi.org/10.1172/JCI186388DS1). However, the fundamental results of the evaluation of the ^68^Ga-DOTA-AC12 probe failed to meet the criteria necessary for clinical application. Consequently, we sought to optimize the molecule by generating an ABY structure, namely, RESCA-B7H3-BCH (Figure 1B).

In our redesign, the N-terminus of the ABY structure was modified using 1-amino-3,6,9,12-tetraoxapentadecan-15-oic acid (PEG4). Additionally, we introduced 2 units of 6-aminohexanoic acid (Acp) at the C-terminus. Then, we used the bifunctional coupling agent H3RESCA-TFP instead of DOTA to modify the probe. The molecular weight and mass dose of ^68^Ga-B7H3-BCH were 7,269 g/mol and 7,400 GBq/kg, respectively, and it exhibited a high specific activity of up to 53.3 GBq/μmol. As a result, pharmacokinetics analysis revealed that the elimination half-life of [^68^Ga]Ga-B7H3-BCH increased from 10.31 minutes to 28.34 minutes (Figure 1C vs. Figure 1D). By extending the plasma half-life of the probe’s distribution phase, its rapid clearance was reduced, and its uptake at the target site was increased. Studies of probe distribution in normal mice demonstrated that, compared with ^68^Ga-DOTA-AC12, [^68^Ga]Ga-B7H3-BCH exhibited a marked reduction in renal uptake, with renal uptake peaks decreasing by approximately 5-fold (721.7% ± 22.0% injected dose per gram [ID/g] vs. 160.8% ± 12.7% ID/g) and exhibiting a rapid decrease over time; these results represented a substantial improvement over the unmodified structure (Figure 1, E and F, and Supplemental Tables 1 and 2). Moreover, the process of labeling the probe with H3RESCA-TFP was considerably milder, requiring only room temperature to achieve extremely high labeling efficiency and radiochemical purity (Supplemental Table 3); this process can prevent potential damage to the ABY structure. The improved synthesis method and complete structural formula of RESCA-B7H3-BCH are shown in Supplemental Figures 2 and 3, and the comprehensive quality control results are shown in Supplemental Figure 4.

### Affinity testing and enhanced PET imaging of the [^68^Ga]Ga-B7H3-BCH radiotracer.

[^68^Ga]Ga-B7H3-BCH exhibited good stability in vitro after a series of modifications (Supplemental Figure 6). Binding affinity assays revealed that this radiotracer had a slightly greater binding affinity (Kd: 4.5 nM) than did ^68^Ga-DOTA-AC12 (Kd: 8.3 nM) (Figure 1, G and H). Further analysis via surface plasmon resonance (SPR) revealed that RESCA-B7H3-BCH had an equilibrium dissociation constant of 6.91 nM (Supplemental Figure 5). Moreover, we noted a substantial reduction in nonspecific [^68^Ga]Ga-B7H3-BCH uptake by most organs (Figure 1, E and F). Through radiation dose estimation, we determined that [^68^Ga]Ga-B7H3-BCH had an effective dose of only 1.19 × 10^–2^ mGy/MBq, which was approximately one-third that of the effective dose of ^68^Ga-DOTA-AC12 (3.3 × 10^–2^ mGy/MBq) (Supplemental Tables 4 and 5). In particular, the radiation dose for the kidney as a single organ decreased from 1.87 mGy/MBq to 0.59 mGy/MBq, which was a safe range. This decrease markedly improved the safety and applicability of the radiotracer in various clinical settings.

Head-to-head micro-PET/CT imaging also demonstrated the superiority of [^68^Ga]Ga-B7H3-BCH in a human lung cancer xenograft model derived from B7H3-transfected H1975 cells; these cells were confirmed by IHC to exhibit high B7H3 expression (Figure 1J). During the 2-hour dynamic imaging session, [^68^Ga]Ga-B7H3-BCH showed a markedly greater uptake at the tumor site than did ^68^Ga-DOTA-AC12 did (Figure 1I). Additionally, statistical analysis revealed that the tumor-to-nontumor (T/NT) tissue maximum standardized uptake value (SUVmax) ratio was greater for [^68^Ga]Ga-B7H3-BCH (7.43 vs. 4.12 at 2 hours), indicating a higher target-to-background ratio (TBR). Furthermore, the TBR peaked at approximately 1 hour and gradually decreased thereafter, providing a reference for determining the optimal imaging time points for subsequent clinical translational imaging studies (Figure 1K).

### Functional evaluation of the [^68^Ga]Ga-B7H3-BCH radiotracer.

**Figure 2:**
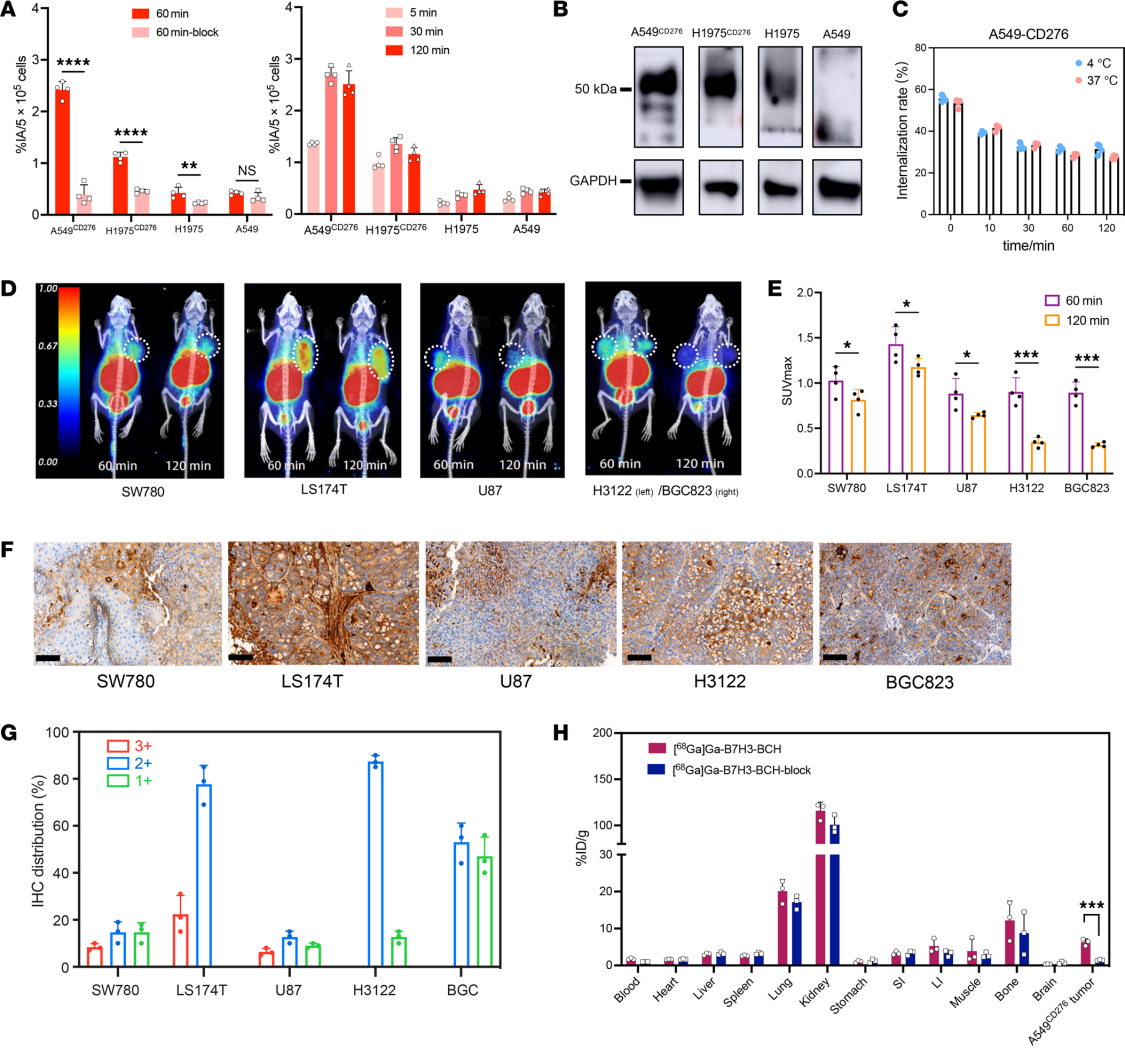
Functional testing of [^68^Ga]Ga-B7H3-BCH radiotracer. (**A**) Cellular uptake and inhibition uptake of [^68^Ga]Ga-B7H3-BCH in B7H3-transfected and untransfected human lung cancer cells at different time points. Data are presented as the mean ± SD (*n* = 4). NS, *P* > 0.05; **P < 0.01 and ****P < 0.0001. (**B**) Expression of B7H3 protein in 4 cell lines by Western blot analysis. GAPDH was used as the loading control. (**C**) Cellular internalization of [^68^Ga]Ga-B7H3-BCH in A549^CD276^ cells. (**D**) PET/CT imaging of 5 different xenograft models — SW780, LS174T, U87, H3122, and BGC823 tumors at 1 and 2 hour after injection. (**E**) Statistical analysis of SUVmax over time for the tumor ROI across various time points. Data are presented as the mean ± SD (*n* = 4). *P < 0.05 and ***P < 0.001. (**F**) IHC staining of the 5 tumor slices (scale bars: 100 μm). (**G**) Grading of IHC regions in 5 tumor sections, classified by staining intensity into B7H3 3+, B7H3 2+, and B7H3 1+. Statistical analysis was conducted on the basis of the proportion of B7H3 expression intensity across different regions. (**H**) Biodistribution and inhibited biodistribution of [^68^Ga]Ga-B7H3-BCH in an A549^CD276^ tumor model. Data are presented as the mean ± SD (*n* = 3). ***P < 0.001, by 1-tailed Student’s *t* test.

[^68^Ga]Ga-B7H3-BCH had highly stable affinity for the B7H3 protein. Its biomolecular targeting efficacy was verified through in vitro cellular assays (Figure 2A). B7H3-transfected A549^CD276^ and H1975^CD276^ cells were engineered and subsequently characterized by Western blotting (Figure 2B). The accumulation of [^68^Ga]Ga-B7H3-BCH in transfected cells substantially exceeded that in nontransfected cells, and this accumulation could be competitively inhibited by cold precursors. Additionally, the maximum uptake typically occurred at 30 minutes, followed by a decrease at 60 minutes. Furthermore, cellular internalization experiments demonstrated that [^68^Ga]Ga-B7H3-BCH was internalized by cells at a high rate, but it was rapidly effluxed from the cells, achieving dynamic equilibrium by 60 minutes; these results suggested optimal timing for in vivo imaging evaluations (Figure 2C).

PET/CT imaging was performed with [^68^Ga]Ga-B7H3-BCH in various xenograft models, including human bladder cancer, colon cancer, glioma, lung cancer, and stomach cancer xenograft models (Figure 2D). Micro-PET/CT images were obtained 1 and 2 hours after injection. Additionally, 2 patient-derived xenograft (PDX) models, including renal carcinoma and gastric cancer PDX models, were subjected to PET/CT imaging (Supplemental Figures 7 and 8). The images revealed heterogeneous uptake of [^68^Ga]Ga-B7H3-BCH at tumor sites in all the xenograft models, which was consistent with the IHC findings from the tumor sections (Figure 2, E and F). PET/CT imaging and statistical analysis revealed a reduction in [^68^Ga]Ga-B7H3-BCH uptake by tumors at 2 hours compared with that at 1 hour, the magnitude of which varied among the different models. Specifically, the H3122 and BGC823 models exhibited a larger decrease than did the SW780, LS174T, and U87 models did (Figure 2E). Moreover, a detailed comparison of the IHC staining intensities revealed diverse B7H3 expression levels across the 5 models. Notably, the SW780, LS174T, and U87 models exhibited regions with strong B7H3 positivity (B7H3 3+), whereas the H3122 and BGC823 models lacked strong B7H3 positivity but still maintained an overall score (Figure 2G); this potentially accounts for the brief retention times of the [^68^Ga]Ga-B7H3-BCH probe within these tumor sites.

### Preclinical preparation and safety evaluation of the [^68^Ga]Ga-B7H3-BCH radiotracer.

A key component of our preclinical studies involved the use of the A549^CD276^ xenograft mouse model to investigate both the biodistribution and blocking ability of the [^68^Ga]Ga-B7H3-BCH radiotracer. The data that were collected 1 hour after injection revealed a marked increase in tracer uptake at the tumor sites compared with that in the blocked group (Figure 2H). Moreover, most organs in the blocked group exhibited reduced uptake, suggesting the potential for residual nonspecific uptake. Additionally, on the basis of the in vivo distribution of [^68^Ga]Ga-B7H3-BCH in mice, the effective radiation dose in humans was calculated to be 1.19 × 10^–2^ mGy/MBq (Supplemental Table 5), which is below the FDA’s imposed limit for research purposes (29). The safety of the [^68^Ga]Ga-B7H3-BCH radiotracer was thoroughly evaluated by toxicological testing. Compared with control mice, normal mice that were administered an overdose of [^68^Ga]Ga-B7H3-BCH (37 MBq/per mouse, *n* = 5) showed no marked change in body weight, and tests revealed normal liver function and blood indicators as well as standard hematological parameters (Supplemental Figure 9). Histopathological examination by H&E staining was conducted 10 days after injection, and the results revealed no pathological changes in major organs relative to those of the controls (Supplemental Figure 10).

### Clinical translation study of the [^68^Ga]Ga-B7H3-BCH radiotracer.

**Figure 3:**
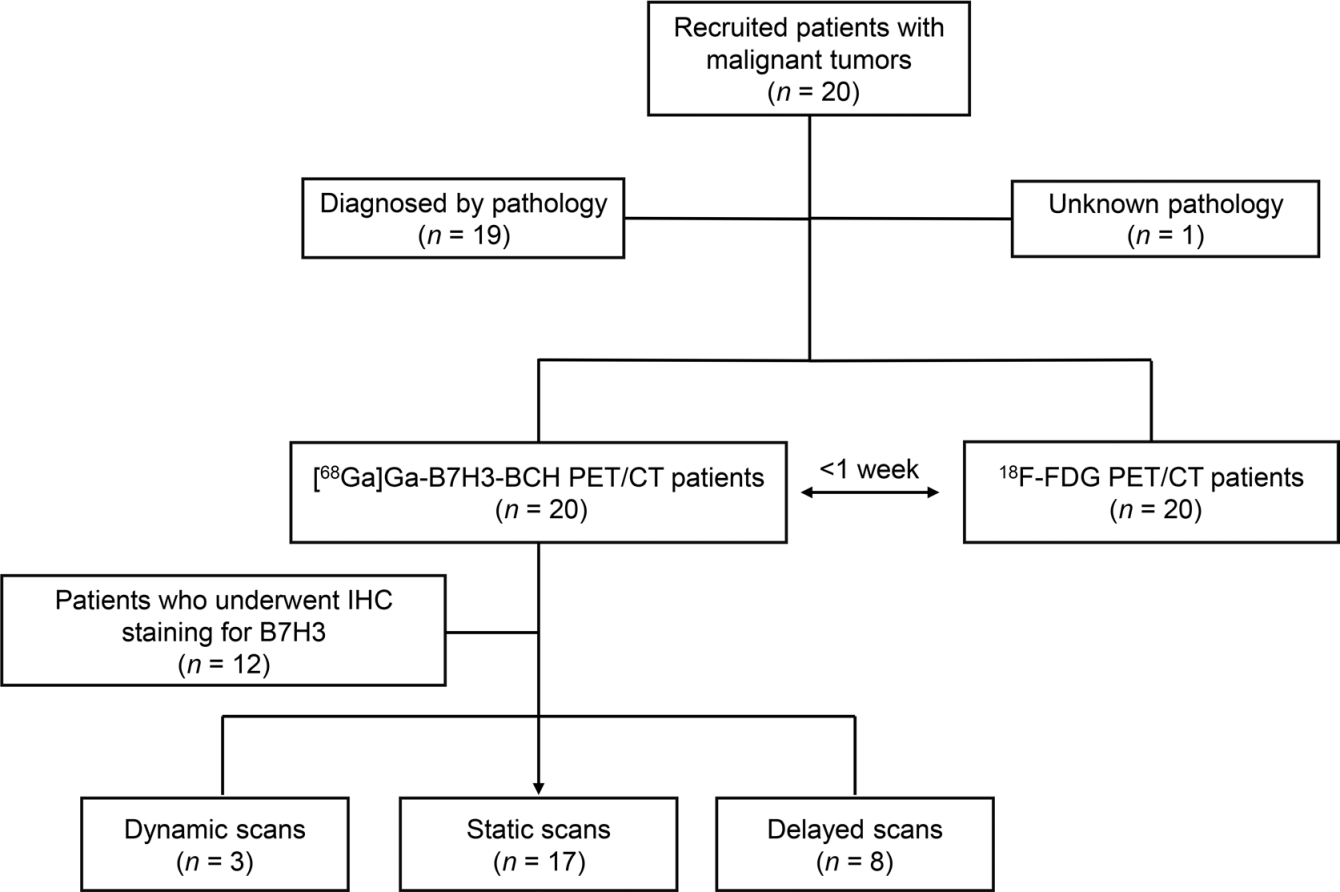
Flow diagram of the clinical study design and scanning methods.

This clinical study included 20 patients with various types of malignant tumors. Detailed participant information is provided in Table 1 and Supplemental Table 7. The PET scanning followed the protocols and dynamic reconstruction methods shown in Figure 3 and Supplemental Figure 11. Each scanning session lasted approximately 5 minutes, which was approximately one-quarter of the duration of conventional PET/CT scans; thus, patient comfort during the procedure was increased. Each patient received 1.42 MBq/kg [^68^Ga]Ga-B7H3-BCH, which was approximately one-third of the dose that is typically used in traditional PET exams. The effective radiation dose of ^68^Ga-B7H3-BCH in humans is 7.02 × 10^–2^ mGy/MBq (Supplemental Table 6), which is slightly greater than the effective radiation dose in a mouse model that simulates human exposure (1.19 × 10^–2^ mGy/MBq). Nevertheless, this dose was low, indicating a high degree of radiation safety, and no adverse effects associated with the injection of the radiotracer and PET/CT scanning were observed in any of the enrolled patients.

**Figure 4:**
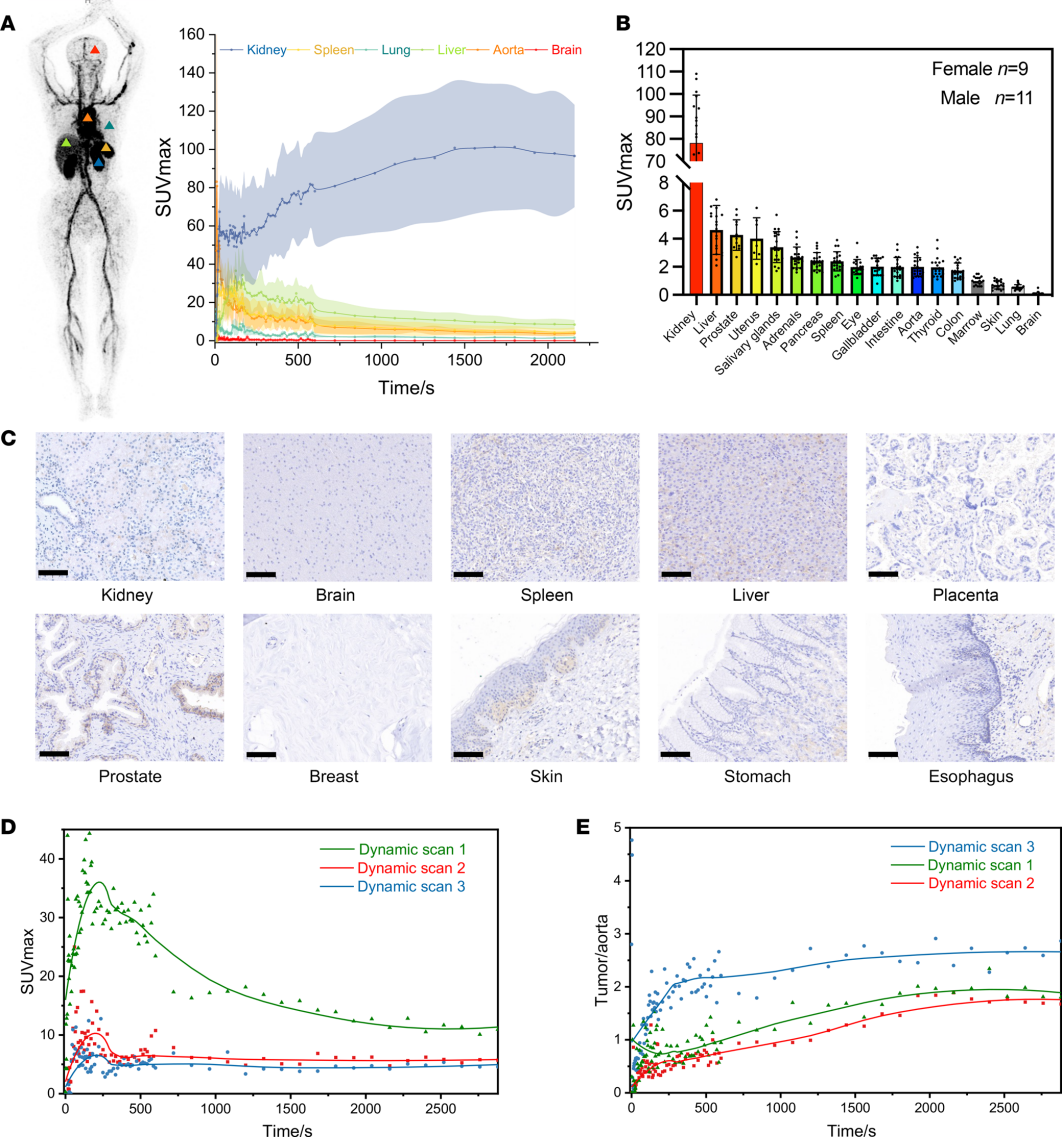
Dynamic PET imaging analysis. (**A**) PET image of 1 patient 30 seconds after injection of the radiotracer, and the dynamic changes in selected organs at 0–35 minutes with SUVmax (*n* = 3). (**B**) Rank ordering of [^68^Ga]Ga-B7H3-BCH uptake in different organs at 50–60 minutes of static PET imaging indicated by SUVmax (*n* = 20). (**C**) IHC staining for B7H3 expression in normal human organ tissue slices (scare bars: 100 μM). (**D**) Dynamic changes in tumor lesions at 0–50 minutes in dynamic PET imaging from 3 representative patients. (**E**) Dynamic changes in the tumor-to-aorta ratio at 0–50 minutes of dynamic PET imaging from 3 representative patients.

Comprehensive multi-time-point, multi-slice dynamic images (including maximum intensity projection [MIP] and axial and coronal views) as well as a complete dynamic reconstruction video of a patient who underwent imaging [^68^Ga]Ga-B7H3-BCH can be found in Supplemental Figure 12 and Supplemental Video 1, respectively. The radiotracer’s temporal distribution curves within tissues were generated via total-body PET/CT (Figure 4A). Notably, kidney uptake gradually increased during the first hour, whereas other organs, such as the spleen, lungs, liver, and aorta, exhibited rapid initial uptake followed by gradual decreases in uptake. Conversely, radiotracer uptake in the brain remained consistently low, suggesting the need for further research on whether [^68^Ga]Ga-B7H3-BCH is hindered by the blood-brain barrier.

Static imaging of all the included patients was performed 50–60 minutes after injection; this time point was initially chosen on the basis of dynamic imaging in mice, which indicated that the highest TBR at the tumor site was observed 50–60 minutes after injection (Figure 1I). Delayed imaging did not yield better results, as the probe was rapidly cleared from the tumor site 1 hour after injection (Supplemental Figure 13). Regions of interest (ROIs) were delineated in 19 major organs, and a statistical analysis of the SUVmax was conducted (Figure 4B). The kidneys continued to show high uptake, although IHC confirmed that normal kidney tissues did not show high levels of B7H3 expression (Figure 4C), indicating that the radiotracer was primarily excreted through the urinary system, and renal retention was observed. The liver, uterus, and prostate demonstrated comparatively higher uptake, and some B7H3 expression within the interstitium of normal liver and prostate tissues was observed by IHC; the uptake by other organs generally corresponded with the IHC results.

Dynamic imaging effectively reveals the temporal distribution for lesions, facilitating the selection of the most suitable imaging time points. As shown in Figure 4D, the SUVmax of the 3 tumor groups peaked between 120 and 150 seconds after the injection of [^68^Ga]Ga-B7H3-BCH, followed by a rapid decrease. Although the dynamic scan 1 showed a gradually slowing reduction, the other 2 measurements were either stable or slowly decreased. The time distribution curves for the tumor-to-aorta ratio across the 3 patients showed a gradual increase within the first 50 minutes, followed by stabilization (Figure 4E). Accordingly, we found that conducting PET/CT imaging between 50 and 60 minutes after injection was consistent with the optimal imaging window.

### Use of the [^68^Ga]Ga-B7H3-BCH for the diagnosis of diverse tumor types.

**Figure 5:**
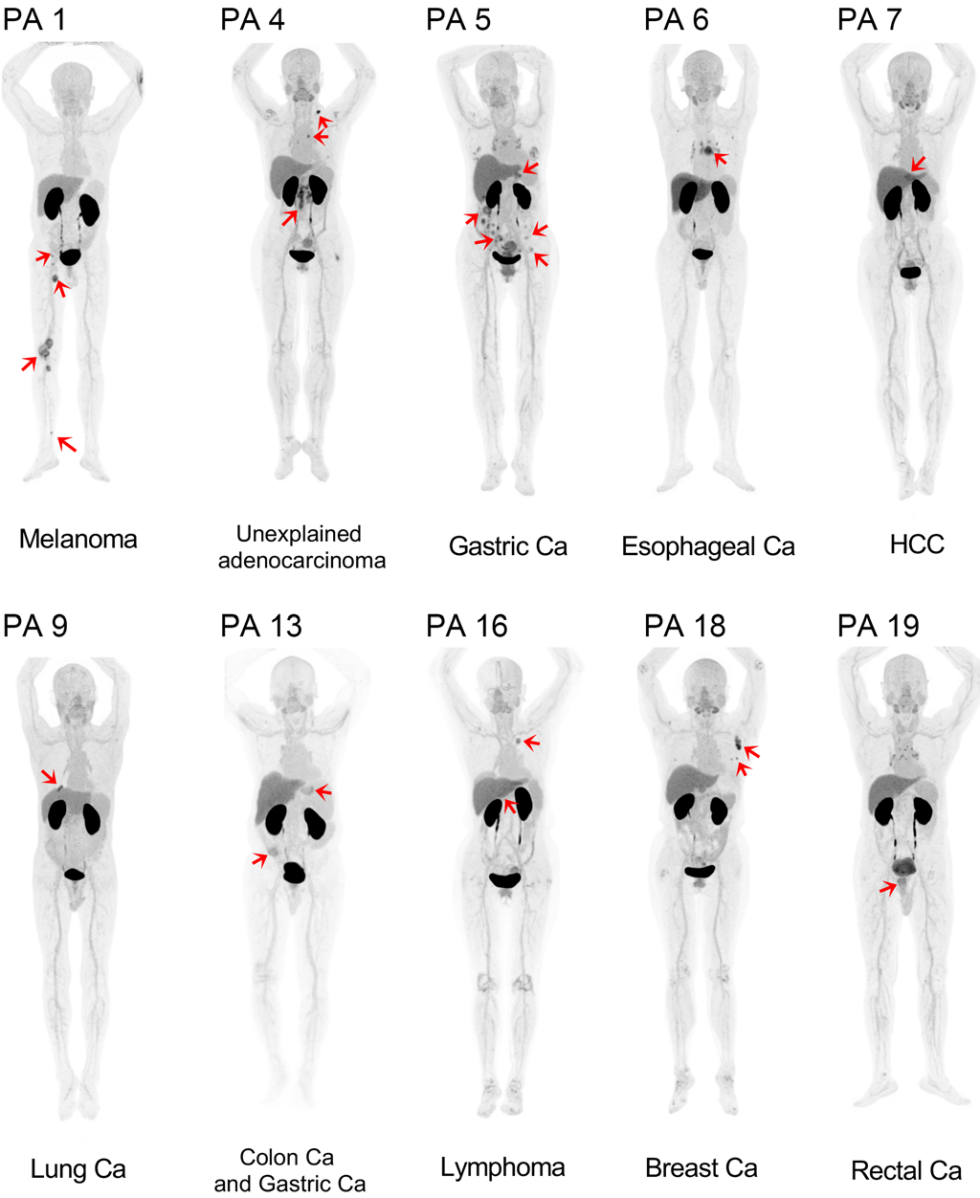
MIP images from [^68^Ga]Ga-B7H3-BCH PET scans of 10 different patients with tumors, with red arrows highlighting both primary and metastatic lesions. Ca, cancer; HCC, hepatocellular carcinoma.

B7H3 is widely expressed by malignant tumors. Thus, 20 patients with various malignancies were evaluated via [^68^Ga]Ga-B7H3-BCH and ^18^F-FDG PET/CT. The patient cohort included 4 patients with lung cancer, 3 patients with melanoma, 2 patients with colon cancer, 2 patients with lymphoma, 2 patients with liver cancer, 2 patients with stomach cancer, 2 patients with esophageal cancer, 1 patient with metastatic lymph nodes of unknown origin, 1 patient with rectal cancer; and 1 patient with breast cancer (Supplemental Table 7). The MIP images shown in Figure 5 highlight the uptake of [^68^Ga]Ga-B7H3-BCH across the 10 different tumor types, revealing varied levels of radiotracer uptake with SUVmax values ranging from 3.7 to 10.7. These images highlight the strong efficacy of [^68^Ga]Ga-B7H3-BCH for the diagnosis of melanoma, breast cancer, lung cancer, gastric cancer, and esophageal cancer, which is principally attributed to the distinct demarcation of lesions against a clear background and a superior signal-to-noise ratio. Furthermore, imaging in a patient with melanoma demonstrated that [^68^Ga]Ga-B7H3-BCH could allow the detailed visualization of both primary and multiple metastatic sites, achieving imaging results comparable to those of ^18^F-FDG (Supplemental Figure 14). In parallel, PET imaging in other patients demonstrated low uptake in some lesions, including in certain patients with lung or colon cancer, indicating tumor heterogeneity in the PET imaging of [^68^Ga]Ga-B7H3-BCH (Supplemental Figure 15). [^68^Ga]Ga-B7H3-BCH shows potential as a specific imaging agent for multiple tumors, as it can be used to resolve misdiagnoses due to the nonspecific uptake of ^18^F-FDG.

### Head-to-head comparison of [^68^Ga]Ga-B7H3-BCH and ^18^F-FDG imaging.

In a study involving 20 patients, 60 tumor lesions were identified, including 21 primary and 39 metastatic sites (verified through histopathology and various imaging modalities). The [^68^Ga]Ga-B7H3-BCH PET identified 51 lesions (85.0%; 18 primary lesions and 33 metastatic lesions), whereas the ^18^F-FDG PET detected 49 lesions (81.7%; 19 primary lesions and 30 metastatic lesions) (Supplemental Table 8). Additionally, 70.0% of these lesions (42 of 60) tested positive for both [^68^Ga]Ga-B7H3-BCH and ^18^F-FDG. A further 9 lesions (15.0%) were positive for [^68^Ga]Ga-B7H3-BCH but negative for ^18^F-FDG, which included 1 primary lesion and 9 metastases. Morphological imaging and follow-up assessments confirmed that merely 3.3% of the lesions (2 of 60) were negative for both tracers, involving 1 metastatic melanoma lymph node and 1 colon cancer lesion.

Comparisons were made between SUVmax and target-to-muscle ratios (TMRs) from [^68^Ga]Ga-B7H3-BCH and ^18^F-FDG PET/CT (Table 2). While the SUVmax values for most tumor types were generally lower on the [^68^Ga]Ga-B7H3-BCH PET/CT compared with the ^18^F-FDG PET/CT, an exception was noted in liver cancer and metastasis of gastric cancer where the uptake was higher. Statistically significant differences were only observed in patients with melanoma and lymphoma. Regarding the TMR, excluding lymphoma, there were no significant differences between the imaging techniques across the various tumor types. Numerically, the TMR values for the [^68^Ga]Ga-B7H3-BCH showed a marked improvement compared with the SUVmax, markedly reducing the discrepancy observed with the ^18^F-FDG uptake across most tumor types.

### Association between [^68^Ga]Ga-B7H3-BCH uptake and B7H3 expression.

**Figure 6:**
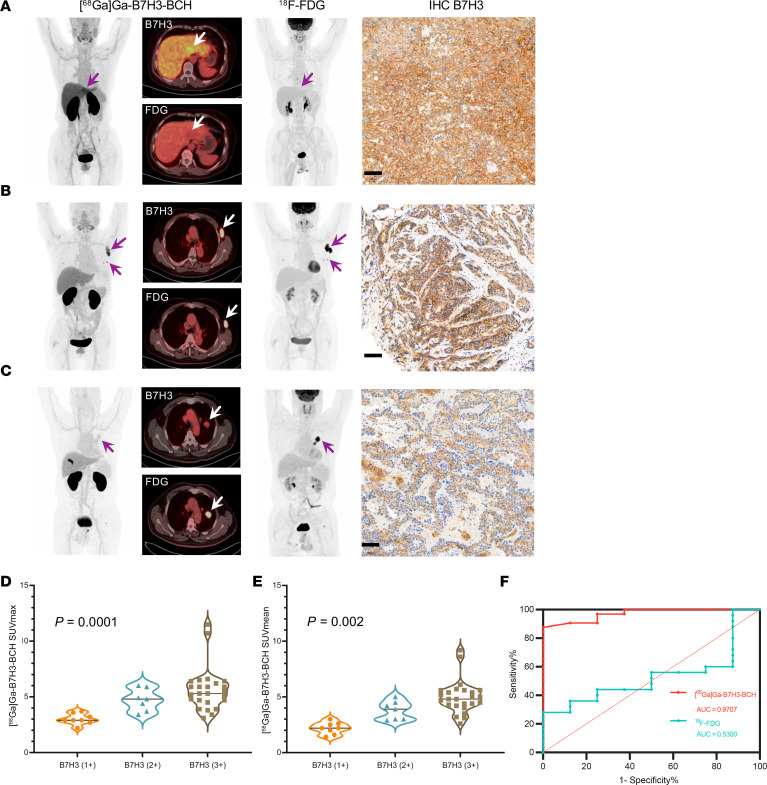
Correlation between PET/CT imaging and B7H3 protein expression. (**A**) Head-to-head [^68^Ga]Ga-B7H3-BCH and ^18^F-FDG PET/CT images and IHC staining of tissue from a patient with liver cancer (this case corresponds to patient [PA] 7 in Figure 5) with high expression of B7H3 (IHC score: 3+) versus low (1+) to moderate (2+) expression levels. IHC score: B7H3 3+. Scale bar: 100 μm. (**B**) Head-to-head PET imaging of a patient with breast cancer (this case corresponds to PA 18 in Figure 5) with moderate-to-high B7H3 expression. IHC score: B7H3 2+. Scale bar: 100 μm. (**C**) Head-to-head PET imaging of a patient with lung cancer with low B7H3 expression levels. IHC score: B7H3 1+. Scale bar: 100 μm. (**D** and **E**) Box plots depicting the SUVmax and SUVmean of [^68^Ga]Ga-B7H3-BCH for all 40 lesions in 12 patients with B7H3 3+, B7H3 2+, or B7H3 1+ expression levels by IHC staining. Statistical significance was determined using a hypothesis test on a linear mixed-effects model (*P* < 0.005 was considered significant). (**F**) ROC curve illustrating the sensitivity and specificity of [^68^Ga]Ga-B7H3-BCH and ^18^F-FDG in evaluating B7H3 expression.

We obtained 12 pretreatment pathological biopsy samples from primary tumor sites in 12 patients, including 2 patients with colon cancer, 2 patients with lung cancer, 2 patients with liver cancer, 2 patients with stomach cancer, 1 patient with esophageal cancer, 1 patient with breast cancer, 1 patient with melanoma, and 1 patient with a metastatic lymph node of unknown origin. Then, we performed IHC staining for the B7H3 protein on these samples. Static imaging with [^68^Ga]Ga-B7H3-BCH demonstrated high uptake in a lesion in a patient with well-differentiated hepatocellular carcinoma, and this high uptake was associated with strong positive IHC staining (B7H3 3+) that was observed in the lesion biopsy (Figure 6A). Furthermore, we observed high uptake in the lesion of a patient with breast cancer, and IHC staining revealed a B7H3 expression level of B7H3 2+ (Figure 6B); however, we observed lower uptake in the lesion of a patient with lung cancer with weak B7H3 expression (B7H3 1+) (Figure 6C). We then performed statistical analysis comparing all the measurable lesions with the results of IHC staining of primary tumor cells; all the lesions that were analyzed were confirmed to be metastases or primary sites by 3 nuclear medicine physicians who were experienced with CT and ^18^F-FDG PET/CT.

The uptake of [^68^Ga]Ga-B7H3-BCH in tumors with B7H3 3+ and B7H3 2+ expression was markedly greater than that in tumors with B7H3 1+ expression (SUVmax: 5.6 ± 1.9 vs. 4.7 ± 1.0 vs. 3.0 ± 0.5). We identified a significant positive correlation between [^68^Ga]Ga-B7H3-BCH uptake and B7H3 expression levels (Figure 6, D and E; *P* < 0.005). In addition, ^18^F-FDG was taken up in large amounts by most lesions, but we noted no significant difference in uptake with ^18^F-FDG as the evaluation criterion (Supplemental Figure 16; *P* > 0.005).

Receiver operating characteristic (ROC) curves were generated to determine the specificity of [^68^Ga]Ga-B7H3-BCH for B7H3-targeted screening. The AUC was 0.9707 for [^68^Ga]Ga-B7H3-BCH at 50–60 minutes of static imaging (95% CI, 92.49% to 100%) and only 0.5300 for ^18^F-FDG at 50–60 minutes of static imaging (95% CI, 32.15% to 73.85%; Figure 6F). These findings demonstrated that [^68^Ga]Ga-B7H3-BCH PET imaging has high specificity for the clinical detection of the B7H3 receptor. When an SUVmax of 3.85 was set as the cutoff to discriminate tumors with B7H3 3+ or B7H3 2+ expression via [^68^Ga]Ga-B7H3-BCH PET/CT imaging, the sensitivity and specificity were 84.38% (95% CI, 68.25%–93.14%) and 100.0% (95% CI, 67.56%–100%), respectively. Overall, [^68^Ga]Ga-B7H3-BCH PET/CT imaging demonstrated excellent sensitivity and specificity for the detection of B7H3-expressing lesions compared with ^18^F-FDG.

### Imaging advantages and influence of variable B7H3 expression.

**Figure 7:**
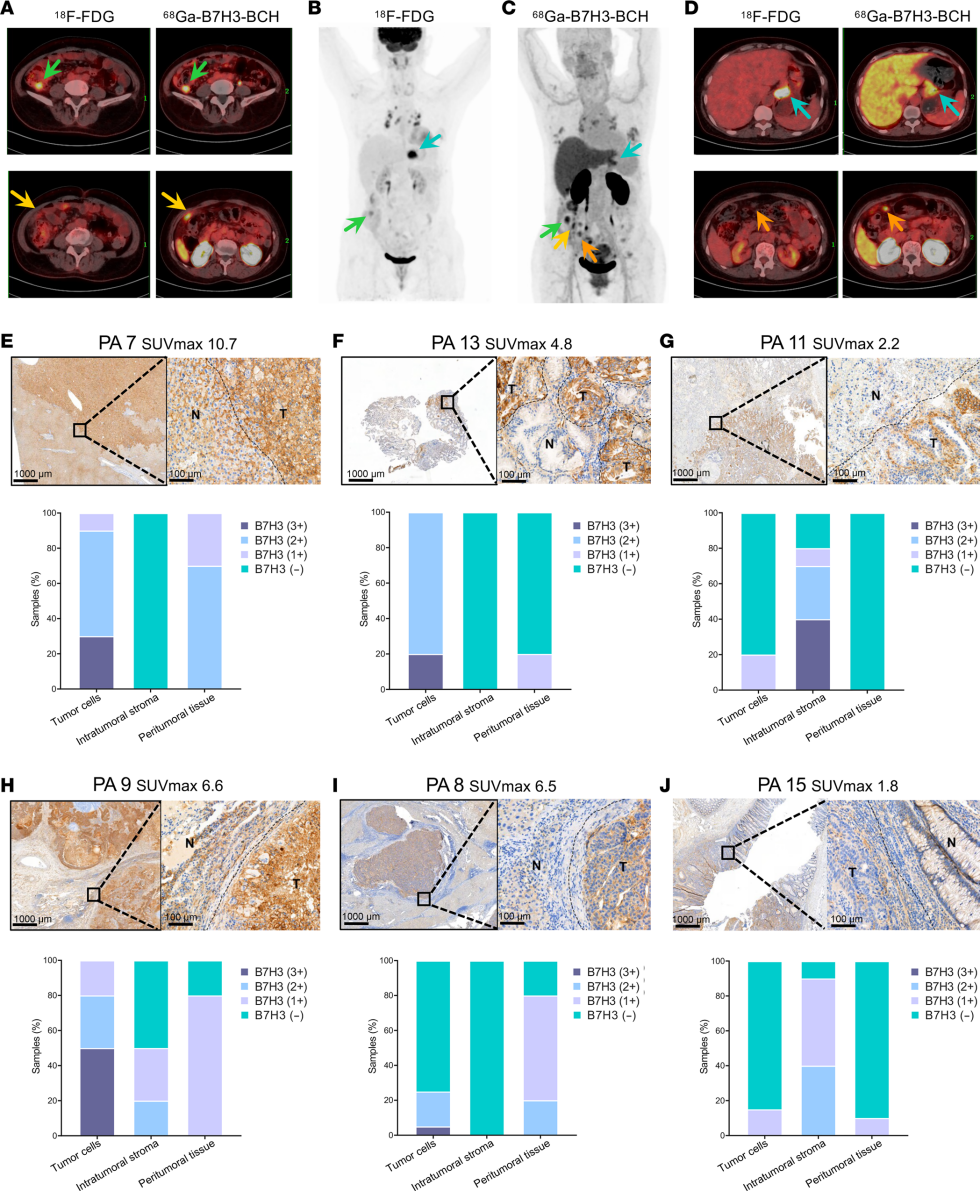
Advantages imaging and influence of variable B7H3 expression. (**A**–**D**) Maximum intensity projection and selected axial PET/CT images of a gastric cancer patient (this case corresponds to PA 5 in Figure 4) with multiple peritoneal metastases, comparing ^18^F-FDG and [^68^Ga]Ga-B7H3-BCH imaging. (**E**) IHC analysis at the tumor margin in a liver cancer biopsy, including tumor cells, intratumoral stroma, and peritumoral tissue with B7H3 expression graded as 3+, 2+, 1+, and negative. “T” represents the tumor region, “N” denotes the nontumor cell area, and dashed lines indicate the boundaries. (**F**–**J**) IHC imaging and data analysis at the tumor margin in a gastric cancer biopsy, 2 lung cancer biopsies, a liver cancer biopsy, and a colon cancer biopsy, respectively.

The methods that can be used to diagnose gastric cancer with peritoneal metastasis by imaging are limited. Despite the high sensitivity of ^18^F-FDG for most peritoneal metastases, early peritoneal gastric cancer metastases, particularly those involving signet ring cell components, may still be missed. Figure 7, A–D, shows a patient with peritoneal metastasis that was missed by ^18^F-FDG, and the patient subsequently underwent [^68^Ga]Ga-B7H3-BCH PET/CT imaging. Both imaging methods revealed marked uptake by the gastric lesion, indicating similar diagnostic effectiveness, but [^68^Ga]Ga-B7H3-BCH imaging revealed multiple peritoneal metastatic sites with high uptake (SUVmax 6.8), in contrast to the low uptake that was observed by ^18^F-FDG imaging (SUVmax <1.5). The lesion near the upper part of the colon, which was initially diagnosed as colonic inflammation by ^18^F-FDG imaging, was considered to be peritoneal metastasis by a nuclear medicine physician who examined the [^68^Ga]Ga-B7H3-BCH imaging results. This result changed the patient’s staging and the chosen surgical approach; this outcome aligned with the primary intention of this study, namely, to develop molecular probes and explore their clinical translation to benefit the patients who were involved in the study.

Numerous studies have shown that B7H3 is not only expressed in tumor cells but also prominently expressed in tumor stromal cells and peritumoral tissues (1, 30, 31). We evaluated IHC staining images of lesions from 12 patients to assess B7H3 expression in 3 distinct areas, namely, tumor cells, intratumoral stroma, and peritumoral tissues. Owing to the limited number of samples, it was not possible to determine whether the distribution of B7H3 expression was associated with specific tumor types. However, by comparing differences in B7H3 distribution with [^68^Ga]Ga-B7H3-BCH uptake, we revealed that B7H3 3+ and B7H3 2+ expression in tumor cells was a necessary condition for achieving SUVmax values above the preestablished cutoff of 3.85 (Figure 7, E, F, H, and I, and Supplemental Figure 17, A–C and F). High B7H3 expression in the tumor stroma alone did not result in [^68^Ga]Ga-B7H3-BCH uptake (Figure 7, G and J, and Supplemental 17, D and E). Moreover, B7H3 expression in the tissues surrounding the tumor did not markedly affect [^68^Ga]Ga-B7H3-BCH uptake. Additionally, high B7H3 expression within both tumor cells and surrounding tissues was associated with increased SUVmax values (Figure 7, E and I). Additional studies with larger samples are needed to confirm these observations.

## Discussion

We have successfully developed an ABY radiotracer that specifically targets the B7H3 receptor protein, and we completed a series of studies ranging from basic research to clinical translation. [^68^Ga]Ga-B7H3-BCH has high affinity, good stability in vitro and in vivo, satisfactory pharmacokinetic parameters, and excellent safety. Total-body PET/CT full dynamic imaging revealed the temporal distribution of the radiotracer in critical human organs and the optimal timing for lesion visualization. PET/CT images showed the specific diagnostic capabilities of [^68^Ga]Ga-B7H3-BCH for various malignancies, particularly its advantages in the diagnosis of well-differentiated hepatocellular carcinoma and gastric cancer peritoneal metastasis. This radiotracer exhibits high specificity and sensitivity in detecting B7H3 expression, making it suitable for noninvasive exploration of primary and metastatic lesions throughout the body.

Previous studies involving B7H3-targeted radiotracers have primarily used monoclonal antibodies as carriers (17, 18). In contrast, ABY-based probes can rapidly accumulate at target sites and are quickly cleared from the body, facilitating the use of short-lived radionuclides for imaging purposes. A prior study utilized ^99m^Tc-labeled AC12 to create single-photon imaging probes for use in foundational experiments (28). However, the limitations inherent to SPECT imaging technology prevented the achievement of optimal imaging outcomes. Using positron-emitting radionuclides to label ABYs and leveraging the latest advances in high-precision PET/CT imaging technology can achieve superior imaging results, increasing the potential for the use of these tools in clinical application. In this study, clinical translation of the total-body PET/CT scanner resulted in superior imaging outcomes. For our clinical investigation, we utilized a 2 meter total-body PET scanner, which offers a sensitivity approximately 15–68 times greater than that of traditional PET/CT (32–34). Moreover, the comprehensive dynamic scanning capability of the scanner facilitated initial investigations into the in vivo distribution and kinetics of [^68^Ga]Ga-B7H3-BCH, enabling the identification of optimal scanning periods. These capabilities have marked benefits for the development of radiopharmaceuticals.

An effective radiotracer must not only exhibit high affinity but also demonstrate stability in vivo, balanced metabolism, and, importantly, optimal tissue uptake, retention, and clearance times to achieve the highest TBR. The modified RESCA-B7H3-BCH, which was improved with PEG4 and Acp, meets these criteria. We have considerable experience with the development of human epidermal growth factor receptor 2–targeted (HER2-targeted) affinity probes and their clinical translation (35). These modifications improve circulation time, increase water solubility and stability, reduce radiolytic degradation, and minimize the potential damage from high-temperature labeling processes due to the incorporation of a RESCA moiety. These modifications also reduce renal uptake and expedite excretion. Finally, the modified [^68^Ga]Ga-B7H3-BCH allows the use of a lower radiation dose without compromising affinity.

The [^68^Ga]Ga-B7H3-BCH radiotracer successfully demonstrated specific imaging capabilities across a broad spectrum of malignancies. Positive imaging results were confirmed in a diverse array of cancer types, including lung cancer, melanoma, colon cancer, lymphoma, liver cancer, stomach cancer, esophageal cancer, rectal cancer, breast cancer, and metastatic lymph nodes of unknown origin. The instances of negative imaging outcomes were consistently associated with low B7H3 expression within lesions. IHC analysis of samples from 12 patients revealed varying levels of B7H3 expression, ranging from B7H3 1+ to B7H3 3+ expression. The acquisition time of these pathologies and the interval between PET imaging sessions were both within 1 month, and no drug treatment was administered in between, thus ensuring that there would be no changes in the distribution of the B7H3 target. Given the extensive expression of B7H3 across various malignant tissues, [^68^Ga]Ga-B7H3-BCH has potential for use as a broad-spectrum oncologic imaging agent. Importantly, the excellent performance of the probe in imaging well-differentiated hepatocellular carcinoma and gastric cancer peritoneal metastases highlight promising application possibilities that merit exploration. On the other hand, [^68^Ga]Ga-B7H3-BCH PET imaging can noninvasively validate changes in targets repeatedly, which will play a significant role in the future of B7H3-targeted therapies prior to their implementation.

In foundational experiments, we initially assessed PET images and performed IHC analyses of 5 xenograft tumor models, and we observed marked heterogeneity in B7H3 expression within the cells of the individual solid tumors. This variance in B7H3 expression could affect the retention time of imaging probes within tumors, thus influencing the selection of optimal imaging periods and the therapeutic effectiveness of radiopharmaceuticals that are developed using ABYs. We will continue to investigate the effect of these differences on imaging protocols. In our clinical research, notable effects of differences in B7H3 expression, as determined by tumor IHC analysis, were observed in the imaging results. Specifically, differences in B7H3 expression among tumor cells, within the tumor interstitium, and peritumoral tissues led to variable uptake of the probe at tumor sites. Our preliminary conclusions suggest that differences in B7H3 expression among tumor cells markedly influenced the uptake of [^68^Ga]Ga-B7H3-BCH, with differences within the tumor interstitium having the least effect; these findings were inconsistent with our initial expectations. Additionally, B7H3 expression in peritumoral tissues was relatively low and had a minimal effect on imaging. This may suggest that with the use of B7H3-targeted therapies, attention should be focused on the distribution of B7H3 within tumor cells, as high B7H3 expression within the tumor interstitium could mislead clinical evaluations prior to treatment. It would be more appropriate to categorize the expression distribution rather than conflating the 2 expression patterns. Furthermore, we plan to perform a correlation analysis between the staining of pretreatment tissues with B7H3-targeted ADCs and patient outcomes, aiming to substantiate these findings. Moreover, the utility of this radiotracer will be expanded to predict the effectiveness of B7H3-targeted therapies and evaluate potential resistance to these therapies in the future.

The ABY was taken up in excessive amounts by the kidneys, and the ABY revealed a certain level of B7H3 expression in the liver. Consequently, notably high accumulation of [^68^Ga]Ga-B7H3-BCH in normal liver and kidney tissues was observed by PET imaging, which interfered with the detection of certain lesions, making PET imaging of renal cancer particularly challenging. Despite a series of chemical modifications to reduce nonspecific uptake in nontarget organs, the ideal biodistribution has not been achieved. On the other hand, dynamic imaging revealed both rapid uptake and clearance of [^68^Ga]Ga-B7H3-BCH at tumor sites, and delayed imaging did not increase the TBR at these locations. However, the clinical sample size of the multitumor imaging analysis in the study was small, which complicates the performance of more in-depth data analysis. Our future plans involve first improving the retention time of the probe at tumor sites, possibly via modifications such as albumin binding or by extending polyethylene glycol chains to increase the molecular weight of the probe, thereby increasing tumor retention. Moreover, we will select tumor types that demonstrate the best imaging results and have the highest clinical diagnostic value for large-scale clinical trials.

## Methods

### Sex as a biological variable.

Among the 20 patients enrolled in this study, 9 were women and 11 were men. All experimental mice were female. The tumor type modeled in this study is not sex specific. To minimize the influence of sex-related variability, we used mice of the same sex to establish the model. Female mice are generally more docile and less aggressive than male mice, reducing the risk of injuries or stress-related variability caused by fighting during experiments. They are also more suitable for group housing, which improves experimental efficiency. In this study, sex was not considered as a biological variable.

### Cell lines and mice.

Human H1975 and A549 lung cancer cells were purchased from the Stem Cell Bank, Chinese Academy of Science, and these cells were cultured in DMEM (Biological Industries). The H1975^CD276^ and A549^CD276^ cell lines were generated via transfection with the full-length CD276 plasmid (Public Protein/Plasmid Library) and cultured in DMEM (MilliporeSigma) supplemented with 1 μg/mL puromycin (Solarbio Life Sciences). All media were supplemented with 10% FBS and 1% penicillin-streptomycin from Biological Industries. The cells were cultured in a humidified incubator at 37°C with 5% CO_2_.

Female BALB/c nude mice, aged 6–8 weeks, were obtained from Vital River. Approximately 1 × 10^6^ H1975, A549, A549^CD276^, or H1975^CD276^ cells were suspended in 100 μL PBS (Solarbio) and s.c. injected into the flanks of nude mice to establish a xenograft model. After 2–3 weeks, when the tumor volumes reached approximately 0.5–1 cm^3^ in size, the mice were kept under specific pathogen–free conditions and subjected to further experiments. Additionally, 6-week-old female Kunming (KM) mice were purchased from Vital River and used for the pharmacokinetics, biodistribution, and toxicity assays.

### Affinity testing assays.

A SPR experiment was performed to evaluate the binding affinity between the precursor and B7H3 with a Biacore 8K system (Cytiva). In brief, after the activation of the Nanogold sensor chip with 1-ethyl-3-(3-dimethylaminopropyl) carbodiimide/*N*-hydroxysuccinimide, the recombinant human B7H3 protein (14058, Cell Signaling Technology [CST]) was immobilized on the chip surface. Next, different concentrations of the precursor were added at a flow rate of 30 μL/min for 240 seconds, and the SPR signal was recorded. The K_D_, association rate constant (ka), and dissociation rate constant (kd) values were calculated with Biacore Insight Evaluation 3.0.12.15655 (Biacore Insight Evaluation Software).

A radioenzyme-linked immunosorbent assay (radio-ELISA) was conducted to determine the binding affinity between the radiopharmaceutical agent and the CD276 protein (catalog KIT11188, Sino Biological). Specifically, 100 μL CD276 (1 μg/mL) protein diluted with carbonate coating fluid was added to a 96-well microplate (CLS2481-100EA, Corning) and then incubated at 4°C overnight. The next day, the microplate was blocked with 5% skim milk and washed with PBST (0.01 mol/L, pH 7.4) (Solarbio). Then, 50 μL of the radiopharmaceutical agent at different concentrations (0.0037-11.1 MBq/mL, 4 wells/group) was added to the microplate and incubated at 37°C for 2 hours. The radiopharmaceutical agent was discarded, and after 5 washes with PBST, the radioactivity of each well was measured with a γ-counter (Wizard2, PerkinElmer). The one-site total mode in Graph Pad Prism 8 (GraphPad Software) was used to fit the relationship between the molar concentration and radioactivity to calculate the K_D_.

### Radiolabeling.

Both RESCA-B7H3-BCH and DOTA-AC12 were synthesized by Tanzhen Bio, ensuring a chemical purity of more than 95%. [^68^Ga]GaCl3 was obtained from a ^68^Ge/ ^68^Ga generator (maximum production 1.85 GBq, Isotope Technologies Garching [ITG]). ^68^Ga-labeling was performed by heating 2.5 mL 0.05 M HCl solution (370–740 GBq), 160 μL of 1.0 M sodium acetate (Aladdin), and 50 μg RESCA-B7H3-BCH or DOTA-AC12 at 90°C for 15 minutes. Then, the solution was extracted by an activated C18 column (activation by 10 mL ethanol and 10 mL water), and the radiolabeled ligand was eluted by 0.5 mL 80% ethanol aqueous solution. After purification, the radiolabeled ligand was obtained with over 99% radiochemical purity analyzed by radio-HPLC. We were able to obtain 300–555 GBq with a radiochemical yield of approximately 75%. The labeling and quality control of [^68^Ga]Ga-B7H3-BCH were performed in a GLP environment by dispensing a hot cell (NMC Ga-68, Tema Sinergie).

### Pharmacokinetics.

One hundred microliters (2.96 MBq, 53.3 GBq/μmol) of ^68^Ga-DOTA-AC12 or [^68^Ga]Ga-B7H3-BCH was injected into female KM mice (*n* = 5) via the tail vein. Blood was collected from the posterior orbital venous plexus at different time points (1, 3, 5, 10, 15, 20, 30, 45, 60, 90, 120, 180, and 240 minutes) and weighed, and radioactivity was measured with a γ-counter. Additionally, a 1% injection volume was used as the standard (*n* = 5). The results are expressed as the percentage of the ID/g. The 2-phase decay mode in GraphPad Prism software was used to analyze the blood pharmacokinetics by fitting the percentage of the ID/g versus the timing of the tracers, thus simulating the metabolic process of the radiotracer in vivo.

### Biological distribution and radiation dose estimation.

For the biological distribution studies, KM mice and A549^CD276^ tumor–bearing mice were injected with 37 MBq/kg ^68^Ga-DOTA-AC12 (200 μL, 53.3 GBq/μmol) or [^68^Ga]Ga-B7H3-BCH (200 μL, 53.3 GBq/μmol) via the tail vein (*n* = 3). The mice were sacrificed at different time points. For blocking, 1 group of A549^CD276^ tumor–bearing mice (*n* = 3) was coinjected with 200 μg unlabeled precursors. Blood and other major organs, including the heart, liver, spleen, lungs, kidneys, stomach, small intestine, muscle, bone, and brain, were collected and weighed, and radioactivity was measured with a γ-counter. As a standard, 5 samples with an injection dose of 1% were collected, and radioactivity was measured. The results are expressed as the percentage of the ID/g.

The fraction of radioactivity uptake by human tissues was calculated according to the biodistribution results in the KM mice. The time-activity curves for various organs and the whole body were generated, and the AUCs for different organs were calculated with GraphPad Prism (GraphPad Software). OLINDA/EXM software, version 2.2 (HERMES Medical Solutions AB), was used to estimate the radiation dosimetry and effective dose for each organ.

### PET imaging of tumor-bearing mice.

PET/CT imaging of the xenograft tumor model was conducted with a small-animal PET/CT machine (Super Nova PET/CT, PINGSENG). When the tumor volumes reached 0.5–1 cm^3^, the mice were i.v. injected with 200 μL radiotracer (277.5–370 MBq/kg, 53.3 GBq/μmol) for small-animal PET imaging. Continuous dynamic imaging was performed for 1 hour after administration, and additional imaging was performed 2 or 4 hours after administration. Unlabeled precursor (500 μg) was coinjected into mice bearing A549^CD276^ tumors to establish the blocking control group. After CT-AC PET reconstruction, the images were analyzed with Avatar software, and the SUVmax values of the ROIs, including the kidney, heart, muscle, and tumor, were manually mapped.

### Cell uptake and internalization experiments.

H1975, A549, A549^CD276^, or H1975^CD276^ cells were suspended in DMEM and added to 24-well plates (5 × 10^5^ cells/well) 1 night prior to the uptake experiments or internalization experiments. For the uptake experiment, after the medium was removed, the plates were washed once with PBS (0.01 M). Then, 500 μL radiopharmaceutical agents diluted with fresh medium (0.074 MBq/well, *n* = 4, 53.3 GBq/μmol) were added to the plates and incubated with the cells at 37°C for 5 minutes, 30 minutes, 60 minutes, or 120 minutes. Next, the plates were washed twice with PBS, and 500 μL of 1 M sodium hydroxide solution was added to lyse the cells. The hydroxide-lysed suspensions were collected, and their radioactivity was measured with a γ-counter. For the blocking control, 50 μg precursor was coincubated with 500 μL dilution mixture for 60 minutes, followed by the steps described above. As a standard, 5 samples with a dilution of 1% were collected, and their radioactivity was measured.

For the internalization experiment, after 20 minutes of incubation at 37°C and 4°C with a mixture of the tracer (0.074 MBq/500 μL, 53.3 GBq/μmol) and DMEM, the medium was removed, and the cells were washed twice with cold PBS (0.01 M). Subsequently, 500 μL serum-free culture medium was added and incubated with the cells for 0 hours, 0.5 hours, 1 hour, 2 hours, or 4 hours. Then, the dissociated (culture medium), membrane-bound (0.1 M acetic acid wash), and internalization (1 M NaOH lysis) fractions were collected. Finally, the radioactivity of each fraction was measured with a γ-counter, and the internalization rate was calculated.

### Western blotting.

For Western blot analysis, a recombinant rabbit monoclonal anti-CD276 primary antibody (diluted 1:100; 14058, CST), an anti-GAPDH antibody (diluted 1:10,000; A19056, ABCLONAL), and an HRP-conjugated secondary antibody (diluted 1:2,000; AS014, ABCLONAL) were used. The final images were processed with an Amersham Imager 680 system (GE Healthcare).

### Toxicology experiments.

Excessive amounts of the radiopharmaceutical agents (500 μL, 1850 MBq/kg, 53.3 GBq/μmol) were i.v. administered to normal KM mice (*n* = 5). Blood samples were collected from the periorbital vein at 1 hour, 1 day, 2 days, and 7 days after injection, and hematological analysis was performed. On the seventh day of the experiment, the mice were euthanized, and the organs of interest were collected for H&E staining. Five mice from the same batch were injected with 500 μL normal saline and used as a control group.

### IHC analysis.

IHC analysis of B7H3 expression was carried out on a Leica BOND III automated immunostainer (Leica Biosystems) with the Bond Polymer Refine Detection Kit (Leica Biosystems, DS9800). Formalin-fixed, paraffin-embedded (FFPE) tissue sections (4 μm thick) were processed through a series of steps, including deparaffinization, antigen repair, and hydrogen peroxide blocking. The sections were subsequently incubated with the anti-B7H3 primary antibody (diluted 1:100; 14058, CST) for 15 minutes at room temperature. All the slides were independently evaluated by 2 pathologists. Normal tissue samples (negative for tumors) were obtained from FFPE specimens that were archived at the Department of Peking University Cancer Hospital. The interval between biopsy or surgery and PET scanning for all patients ranged from 2 to 30 days. Pathology results were obtained prior to imaging for 4 patients and after imaging for 8 patients. No patients received pharmacological treatment during this period. For each sample, both the staining intensity and the percentage of positive cells within the tumor and intratumoral stroma were independently recorded. The B7H3 staining intensity was categorized as follows: 0 for no staining, 1 for mild membranous staining, 2 for moderate membranous staining, and 3 for strong membranous staining. The histochemistry score (H-score) was calculated by multiplying the percentage of positive cells by the staining intensity, with a maximum score of 300. The samples were further classified into 3 categories on the basis of their H-scores: 1+ (H-score <100), 2+ (H-score 100–200), and 3+ (H-score >200).

### Clinical trial approval and patient eligibility criteria.

The important inclusion criteria for the oncological patients were as follows: age between 18 and 75 years and a malignant tumor diagnosis prior to pharmacological treatment and surgery. The important exclusion criteria included severe impairment of liver and kidney function, pregnancy, or lactation. Twenty patients with malignant tumors were enrolled in this study.

### Total-body PET/CT imaging.

A 194 cm long axial field-of-view (FOV) total-body PET/CT (uEXPLORER, United Imaging Healthcare) was used. A low-dose CT scan was performed before the [^68^Ga]Ga-B7H3-BCH injection, and 3 patients subsequently underwent a total-body dynamic PET scan for 50 minutes after injection of 1.42 MBq/kg [^68^Ga]Ga-B7H3-BCH. The other 17 patients underwent a static PET/CT scan at 50–60 minutes. Among the participants, 8 patients underwent delayed static scans at 120–125 minutes, and among these patients, 3 underwent dynamic scans and 5 had poor lesion visualization or suboptimal imaging outcomes. All the patients underwent an ^18^F-FDG PET/CT scan at 50–60 minutes with 4.00 MBq/kg. Total-body PET/CT with low-dose CT scans (50 mAs, 140 kVp) were performed for the first scan, and ultralow-dose CT scans (5 mAs, 140 kVp) were performed for delayed scanning.

### Total-body PET/CT image reconstruction.

Three reconstructions were performed: a dynamic reconstruction of data from 0–50 minutes, a static reconstruction of data from 45–50 minutes, and a static reconstruction of delayed scans. Dynamic reconstruction included 90 dynamic time frames as follows: 0–30 seconds with 2-second frames, 30–180 seconds with 5-second frames, 180–600 seconds with 14-second frames, and 600–2,280 seconds with 120-second frames. All the time frames were reconstructed with the ordered subset expectation maximization (OSEM) method (4 iterations, 20 subsets), with corrections for the point spread function (PSF) and time of flight (TOF). The dynamic distribution of the ROIs in tumors and major organs was extracted from all 90 frames from 0 to 38 minutes. Static images were reconstructed from list-mode data with vendor-provided software (United Imaging), using an iterative algorithm (20 subsets, 4 iterations) that incorporated TOF information but excluded PSF correction.

### [^68^Ga]Ga-B7H3-BCH biodistribution and dosimetric evaluation in humans.

The activity of ^68^Ga was decay corrected according to the injection time point and normalized to the total activity. Data processing was performed with the vendor-provided software Multi-Modality Workplace (United Imaging). To analyze the biodistribution of [^68^Ga]Ga-B7H3-BCH, ROIs were manually delineated on the maximum cross section of major organs/tissues in a 50- to 60-minute scan. The normal organs/tissues that were selected for volume-of-interest (VOI) analysis included the salivary glands, kidneys, aorta, esophagus, colon, liver, pancreas, intestines, thyroid, spleen, adrenal glands, eyes, gallbladder, skin, lung, and brain. The SUVmax of each ROI was automatically calculated with the vendor’s software and was utilized for analysis and comparison. The SUVmax was defined as follows: *r* is the maximum radioactivity activity concentration (kBq/mL) as measured by the PET scanner within the defined ROI, *a′* is the decay-corrected amount (kBq), and *w* is the weight of the patient (in grams).

### ROC curves.

Tumor tissues were collected from 12 patients and included in the ROC curve analysis. The tumor tissues were initially subjected to IHC staining for the B7H3 protein. According to the expression results, the samples were categorized as B7H3 1+, B7H3 2+, or B7H3 3+, resulting in 32 positive lesions (from 8 patients) and 8 negative lesions (from 4 patients). For each lesion, the SUVmax at 50–60 minutes for [^68^Ga]Ga-B7H3-BCH PET and the SUVmax for ^18^F-FDG PET were analyzed. Owing to the inability to obtain pathological biopsies from all lesions, we assumed in our statistical analysis that the primary and metastatic lesions in the enrolled patients shared equivalent B7H3 expression levels on the basis of their homogeneity, and these SUVmax values were paired with the B7H3 scores of each lesion to generate ROC curves.

### Statistics.

Statistical analysis was performed using GraphPad Prism 8.0 (GraphPad Software) and Origin software (V2018, Microcal). The statistical results validated and met the prespecified primary endpoint of the registered trial. The fluorescence intensity, cellular uptake, blood biochemical parameters, tumor xenograft biodistribution, and other comparative data from 2 independent samples were analyzed by unpaired, 1-tailed Student’s *t* tests. A *P* value of less than 0.05 was considered statistically significant. Organ uptake data in the form of the SUVmax were grouped by sex and age. To compare distributions between samples, continuous variables are presented as the mean ± SD. The SUVmax and TLR of [^68^Ga]Ga-B7H3-BCH and ^18^F-FDG PET/CT were compared using the paired-samples *t* test (normally distributed variables). A hypothesis test on a linear mixed-effects model was performed to assert the differences in the SUVmean and SUVmax values among the 3 groups corresponding to the pathological grades of all lesions. A *P* value of less than 0.005 was considered to indicate statistical significance. The criteria for determining the optimal cutoff value of [^68^Ga]Ga-B7H3-BCH were the point on the ROC curve that was closest to the upper left corner of the unit square and the point that had the highest Youden index (sensitivity + specificity). The total AUC and its 95% CI were calculated. The sensitivity and specificity were calculated as indicators for predicting B7H3 expression.

### Study approval.

All animal experiments were approved by the ethics committee of Peking University Cancer Hospital (approval no. EAEC 2023–18). The clinical study was approved by the ethics committee of Peking University Cancer Hospital (approval no. 2023KT131), and this study is registered with ClinicalTrials.gov (NCT06454955). Written informed consent was obtained from all study participants.

### Data availability.

All relevant data can be found in the article and its supplemental materials, including the Supporting Data Values file.

## Author contributions

LX was responsible for the overall design of the experiment, participated in and completed the experiments, wrote the main manuscript, and provided funding support. YW, LXZ, and LJ performed IHC staining and analysis of the mouse and human tissue sections. YNR participated in and completed the basic experiments. ZW collected and analyzed the patients’ clinical information. NZ, XXM, and HZ provided technical support for the PET/CT imaging equipment, image processing, and clinical diagnosis. ZY and CXH provided the CD276-transfected cells and conducted the Western blot analysis. ZY participated in the experimental design and the writing and revision of the manuscript. All the authors reviewed the manuscript. With regard to the order of the first authors’ names, LX initiated the research, completed most of the experiments, and wrote the majority of the manuscript and is thus listed first; YW performed extensive IHC staining and analysis of sections from patients, which constituted a portion of the content of the article and thus is listed second; YNR primarily conducted the foundational experiments for basic research, playing a key role in the early stages of the study, and thus is listed third; and ZW was responsible for communicating with clinical patients, screening and enrolling participants, and performing some data analysis, and thus is listed fourth.

## Supplementary Material

Supplemental data

ICMJE disclosure forms

Unedited blot and gel images

Supplemental video 1

Supporting data values

## Figures and Tables

**Table 2:**
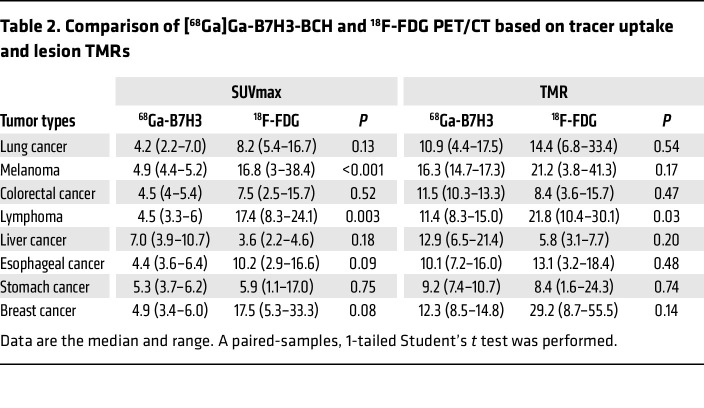
Comparison of [^68^Ga]Ga-B7H3-BCH and ^18^F-FDG PET/CT based on tracer uptake and lesion TMRs

**Table 1:**
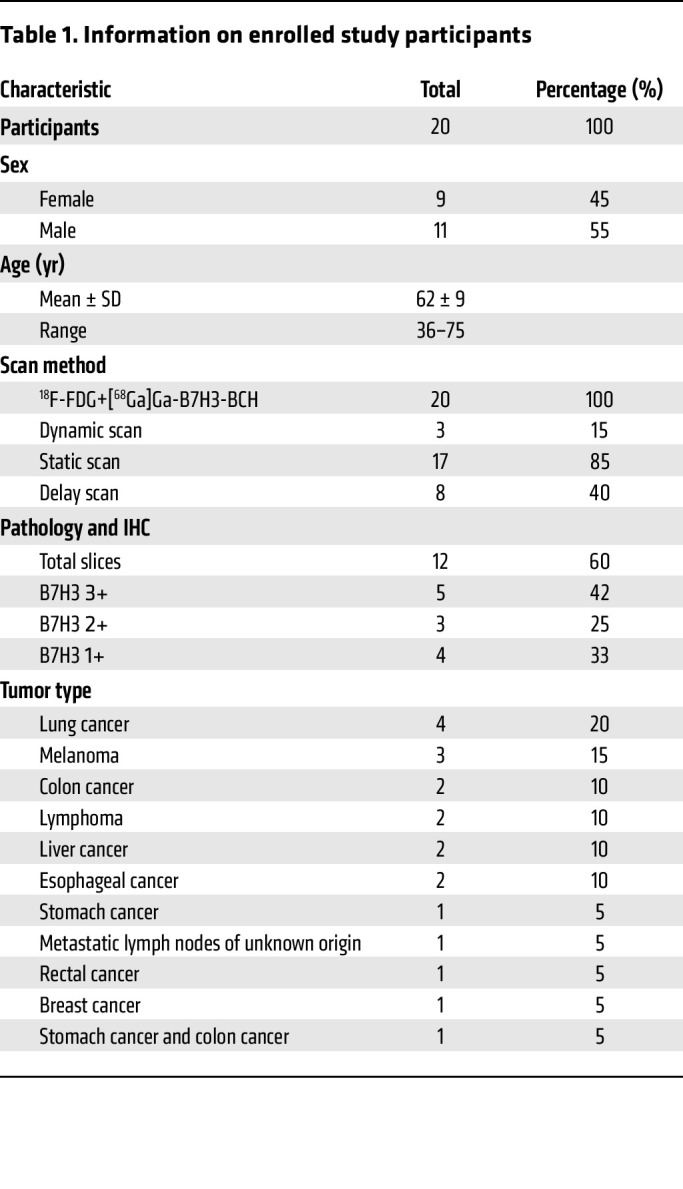
Information on enrolled study participants

## Version 1 01/23/2025

In-Press Preview

## Version 2 03/17/2025

Electronic publication
