# Supplementary material for: A whole-body imaging technique for tumor-specific diagnostics and screening of B7H3-targeted therapies
Source: J Clin Invest. 2025 Jan 23;135(6):e186388. doi: 10.1172/JCI186388 (PMC11910224; doi:10.1172/JCI186388)
Supplement: Supplemental data [file jci-135-186388-s237.pdf]

Supplementary Materials for

A Whole-Body Imaging Technique for Tumor-Specific Diagnostics and Screening of B7-H3-Targeted Therapies

Lei Xia[1]†*, Yan Wu[2]†, Yanan Ren[3]†, Zhen Wang[4]†, Nina Zhou[1], Wenyuan Zhou[1], Lixin Zhou[2], Ling Jia[2], Chengxue He[1], Xiangxi Meng[1], Hua Zhu[1], Zhi Yang[1]*

[1]Key Laboratory of Carcinogenesis and Translational Research (Ministry of Education/Beijing), NMPA Key Laboratory for Research and Evaluation of Radiopharmaceuticals (National Medical Products Administration), Department of Nuclear Medicine, Peking University Cancer Hospital & Institute, Beijing, China.

[2]Key laboratory of Carcinogenesis and Translational Research (Ministry of Education/Beijing), Department of Pathology, Peking University Cancer Hospital & Institute, Beijing, China.

[3]Department of Nuclear Medicine, Affiliated Hospital of Zunyi Medical University, Zunyi, Guizhou, China.

[4]Key Laboratory of Carcinogenesis and Translational Research (Ministry of Education, Beijing), Department of Hepato-Pancreato-Biliary Surgery, Sarcoma Center, Peking University Cancer Hospital and Institute, Beijing, China.

†These authors contributed equally to this work

***Corresponding authors**

Lei Xia, Peking University Cancer Hospital & Institute, 52 Fucheng Rd., Beijing 100142, China. Tel: +86-010-88197852, Email: xialei9012288@126.com

Zhi Yang, Peking University Cancer Hospital & Institute, 52 Fucheng Rd., Beijing 100142, China. Tel: +86-010-88196196, Email: pekyz@163.com

This file includes:

Materials

Figure. S1 to S17

Table. S1 to S8

SUPPLEMENTARY MATERIALS

General

All solvents and chemicals purchased from commercial sources were of analytical grade or higher and could be used without further purification. The final products were characterized by high-resolution mass spectrometry (HRMS) using an AB SCIEX 3200 system (AB SCIEX, Concord, Canada). High-performance liquid chromatography (HPLC) analyses were conducted on a Waters 2695 system (Waters, USA) using COSMOSIL 5C18-MS-II columns (4.60ID×250mm, COSMOSIL, Japan). The solvent gradients used were A: 0.1% TFA in 100% H_2O and B: acetonitrile, with a flow rate of 1 mL/min. The HPLC conditions included a gradient from 32-52% B over 20 minutes, and retention times were reported. The radiochemical purity of ^{68}Ga-labeled compounds was analyzed using a radio-HPLC system equipped with a RP18 10μm (4.6

mm × 250 mm) column (20A, Shimadzu, Japan), under conditions of 32-52% B (0.1% trifluoroacetic acid in acetonitrile) over 20 minutes.

SUPPLEMENTARY FIGURES

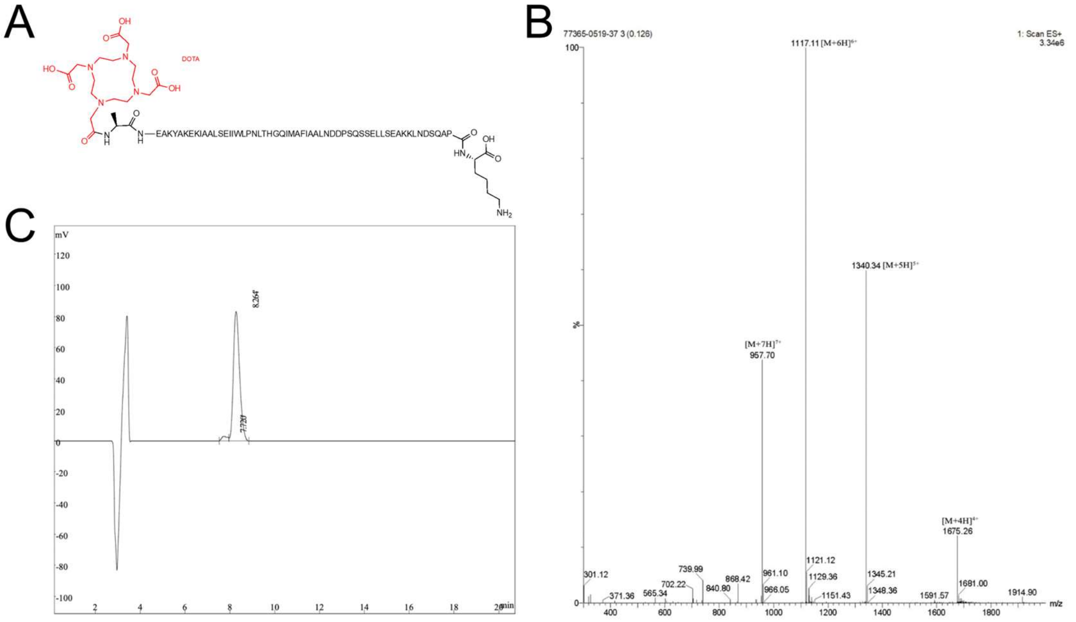

Figure S1. Synthesis of DOTA-AC12. **A** Structure of DOTA-AC12. **B** ICP-MS of DOTA-AC12. **C** HPLC of DOTA-AC12. (Time: 20 min; Wavelength: 220 nm; Flow: 1 mL/min; Mobile phase A: MeCN with 0.1% TFA; Mobile phase B: H_2O with 0.1% TFA; Separation column: Kromasil 100-5C18, 4.6 mm×250 mm, 5-micron TFA, TIPS and water).

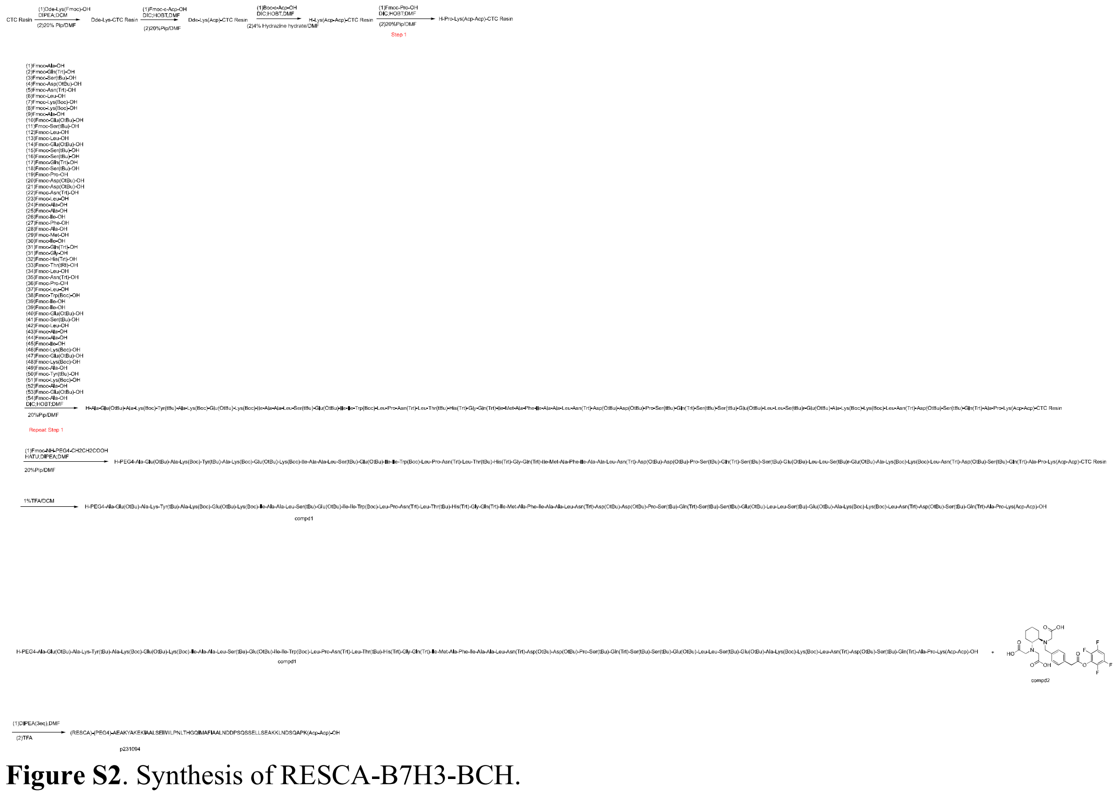

Figure S2. Synthesis of RESCA-B7H3-BCH.

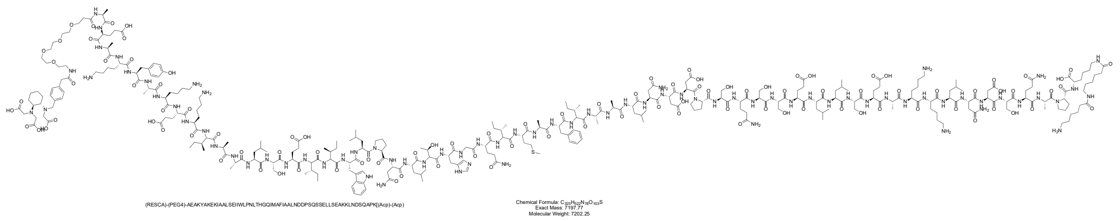

Figure S3. Complete chemical structure of RESCA-B7H3-BCH affibody

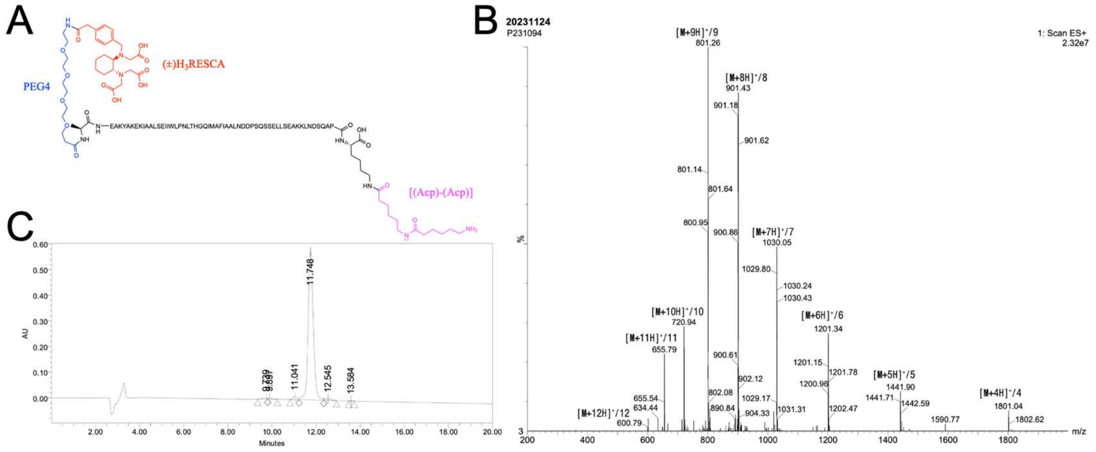

Figure S4. Synthesis of RESCA-B7H3-BCH. **A** Structure of RESCA-B7H3-BCH. **B** ICP-MS of RESCA-B7H3-BCH. **C** HPLC of RESCA-B7H3-BCH. (Time: 20 min; Wavelength: 220 nm; Flow: 1 mL/min; Mobile phase A: MeCN with 0.1% TFA; Mobile phase B: H_2O with 0.1% TFA; Separation column: Kromasil 100-5C18, 4.6 mm×250 mm, 5-micron TFA, TIPS and water).

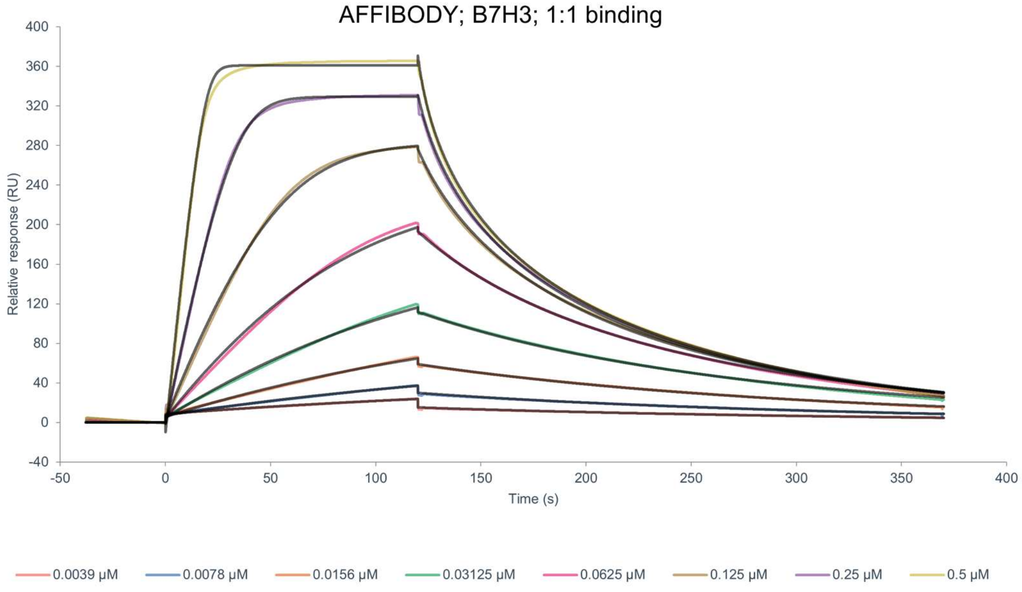

Figure S5 SPR characterization of RESCA-B7H3-BCH.

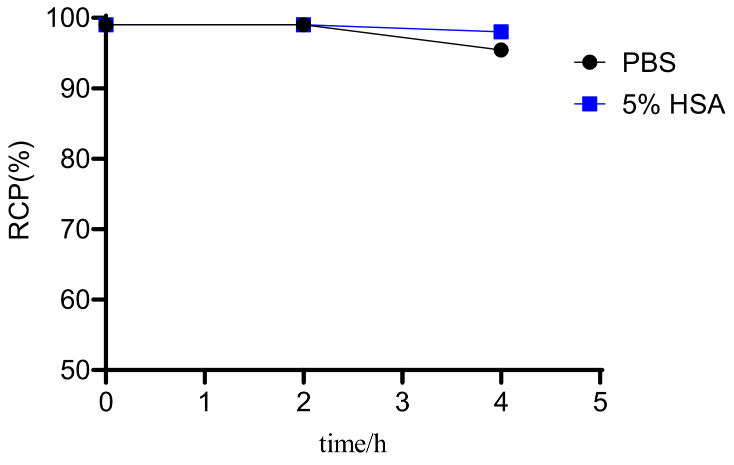

Figure S6 Stability assessment of ^{68}Ga-B7H3-BCH in vitro in PBS and human serum albumin solutions

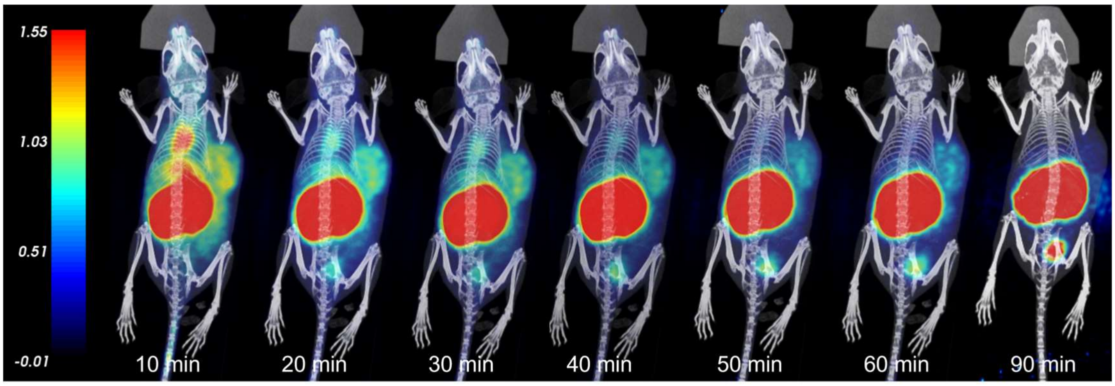

Fig. S7 Micro-PET/CT imaging of ^{68}Ga-B7H3-BCH in renal carcinoma PDX model.

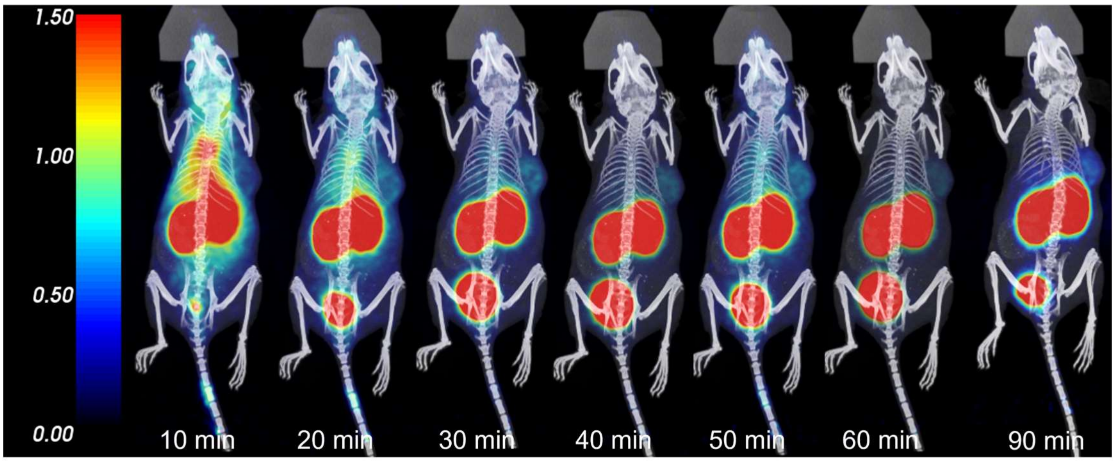

Figure S8 Micro-PET/CT imaging of ^{68}Ga-B7H3-BCH in gastric cancer PDX model.

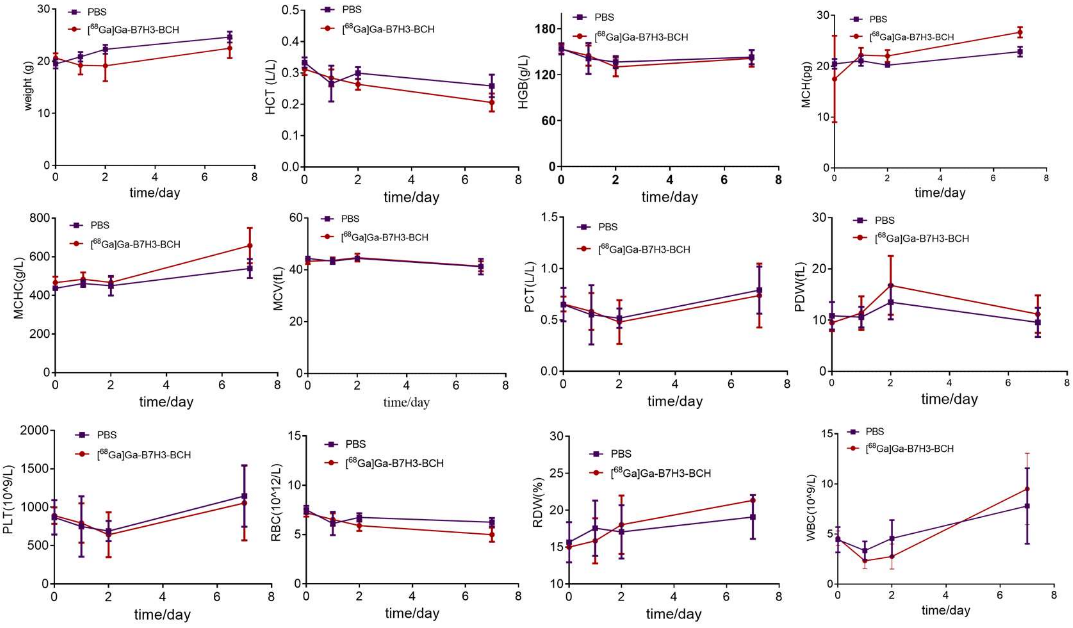

Fig. S9 Toxicological experiment. Complete blood count and liver function tests for the experimental and control groups.

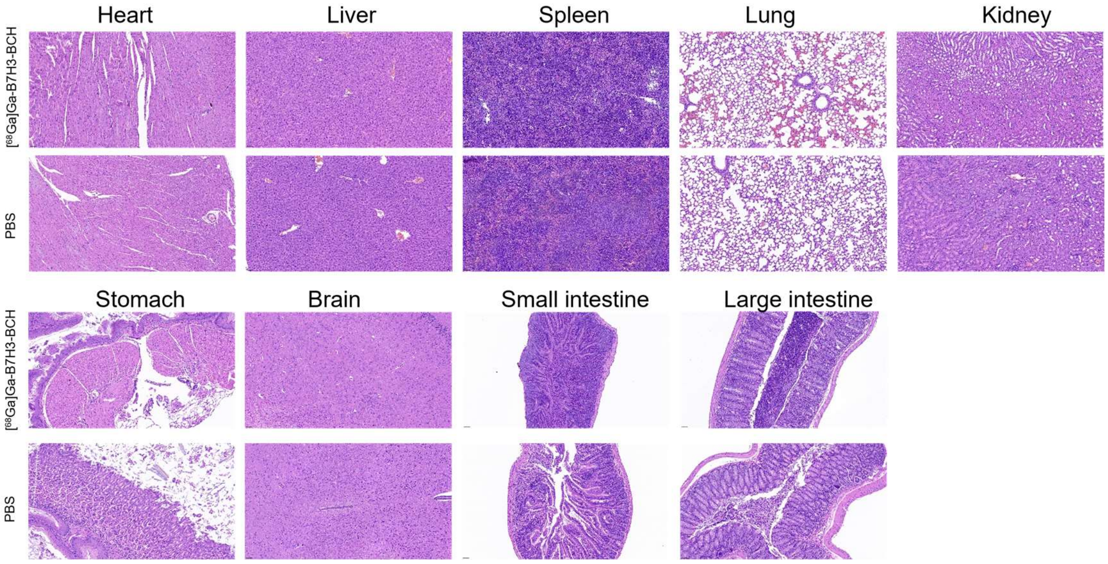

Fig. S10 Toxicity test of ^{68}Ga-B7H3-BCH. **A** H&E staining of main organs taken from test group and control group.

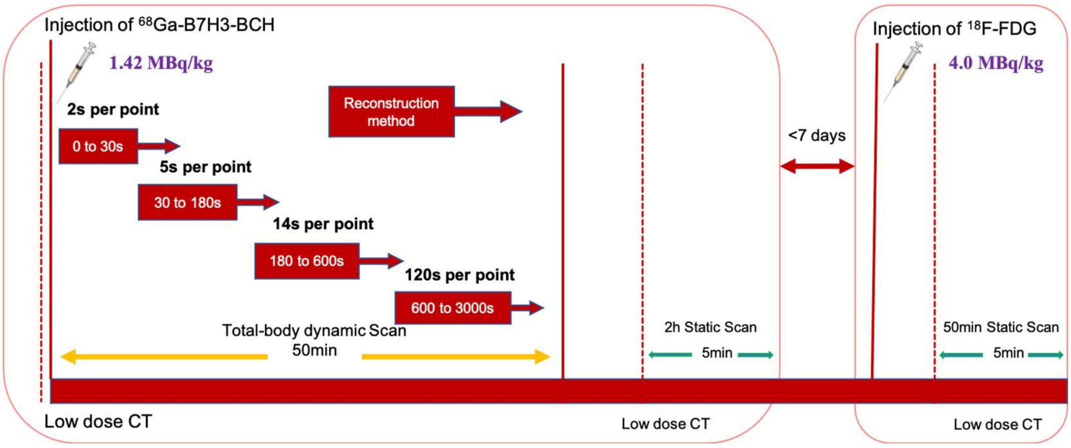

Fig. S11 Dynamic imaging time protocol. A low-dose CT scan was performed before injection of ^{68}Ga-B7H3-BCH. Then, a dynamic total-body PET scan was continued for 50 min and a static scan was performed for 5 min at 2 h. ^{18}F-FDG PET scan was performed with a static scan for 5 min at 50 min with a low-dose CT.

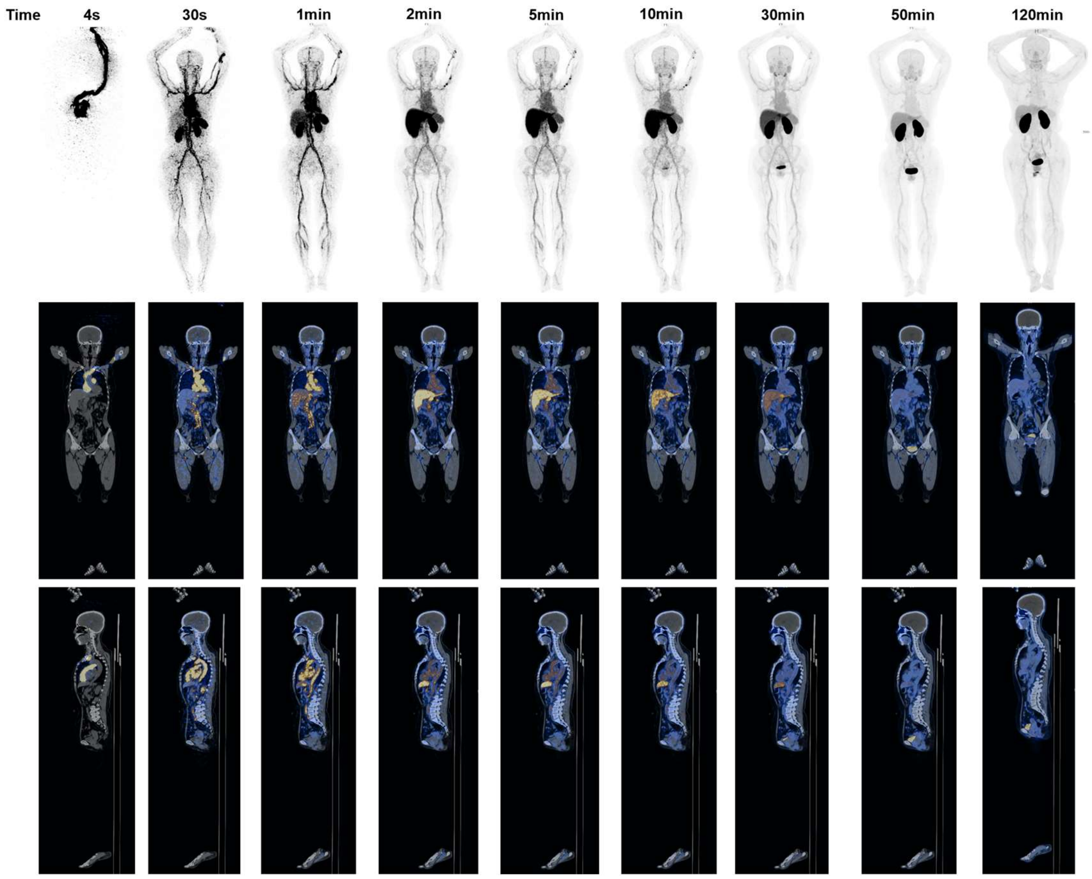

Fig. S12 Results of PET/CT dynamic imaging studies in one patient. Top row indicates ending time of whole-body scan. Decay-corrected anterior maximum intensity projections (MIP) and whole-body coronal and mid-sagitta of PET/CT images at 4s, 30s, 1min, 2min, 5min, 10min, 30min, 50min and 120min (from left to right) after injection of ^{68}Ga-B7H3-BCH in a dynamic imaging patient.

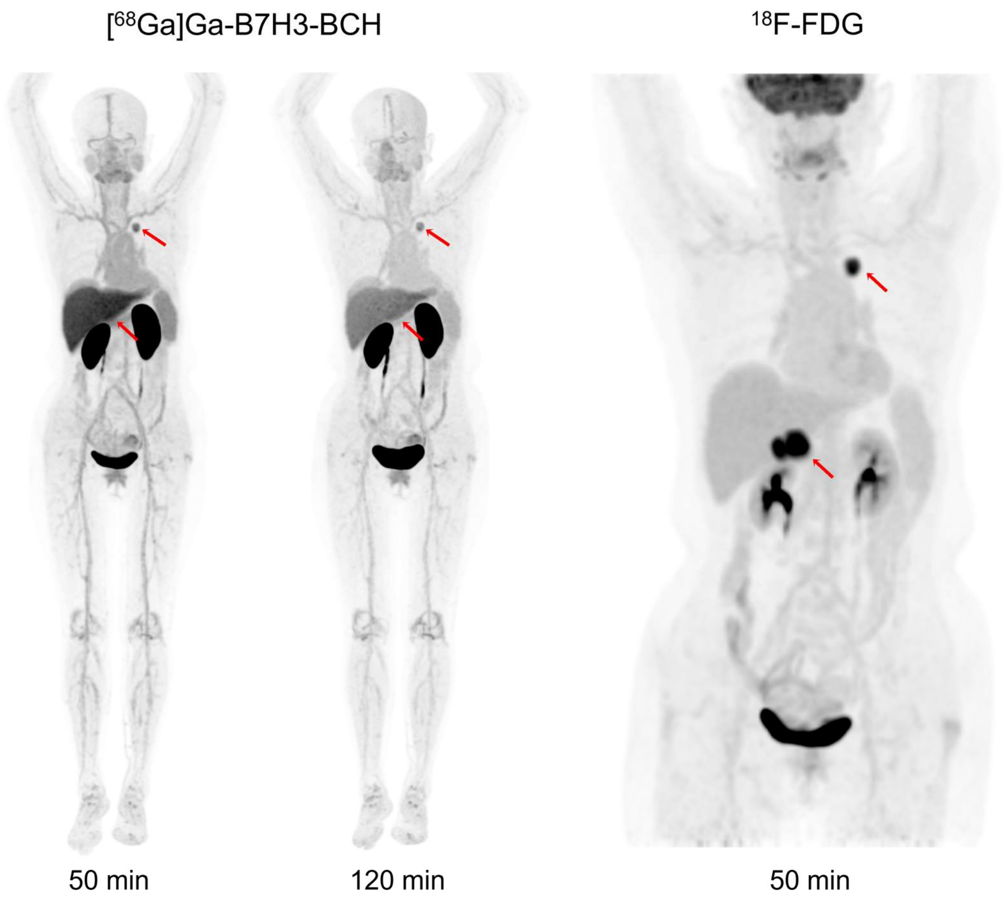

Fig. S13 Maximum intensity projections (MIP) PET imaging of Patient 16 at 50 min and 120min after injection of [^{68}Ga]Ga-B7H3-BCH, and at 50 min after injection of ^{18}F-FDG.

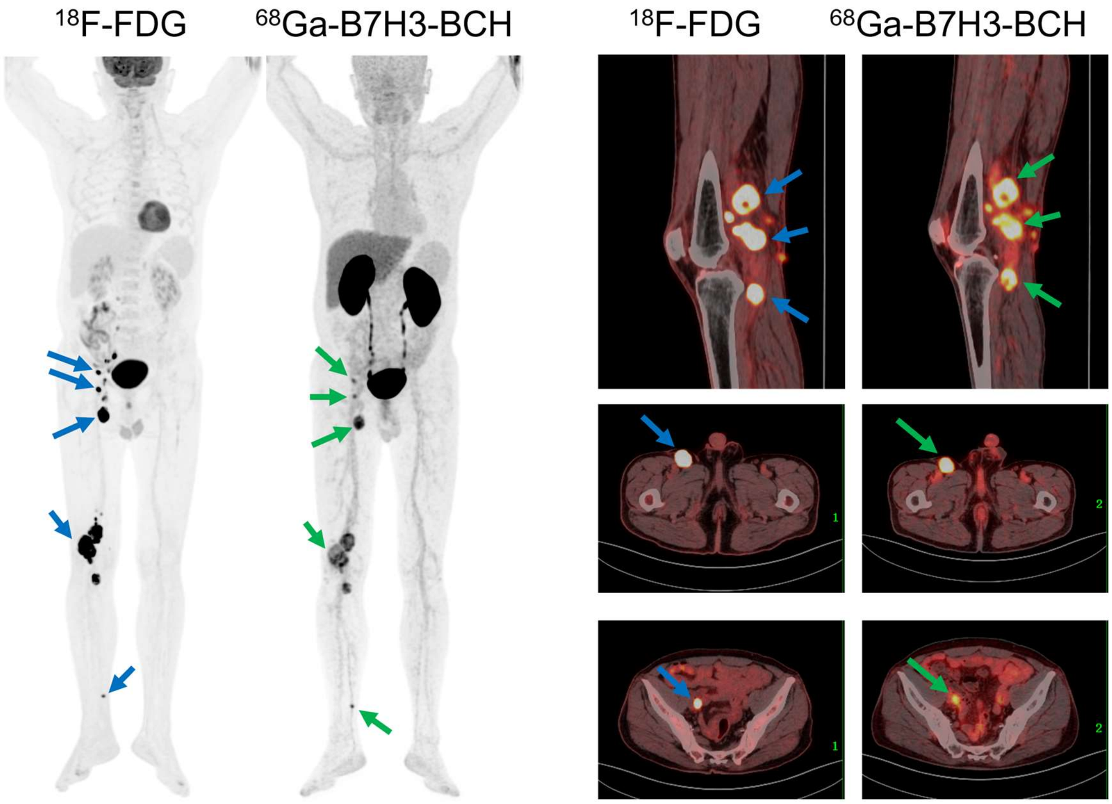

Fig. S14. Representative PET/CT images of a patient with melanoma. Decay-corrected anterior maximum intensity projections (MIP) PET imaging at 60 min after injection of ^{68}Ga-B7H3-BCH, and at 60 min after injection of ^{18}F-FDG. Slice sagittal PET/CT imaging of represent popliteal, inguinal, and para-iliac vascular metastatic lymph nodes.

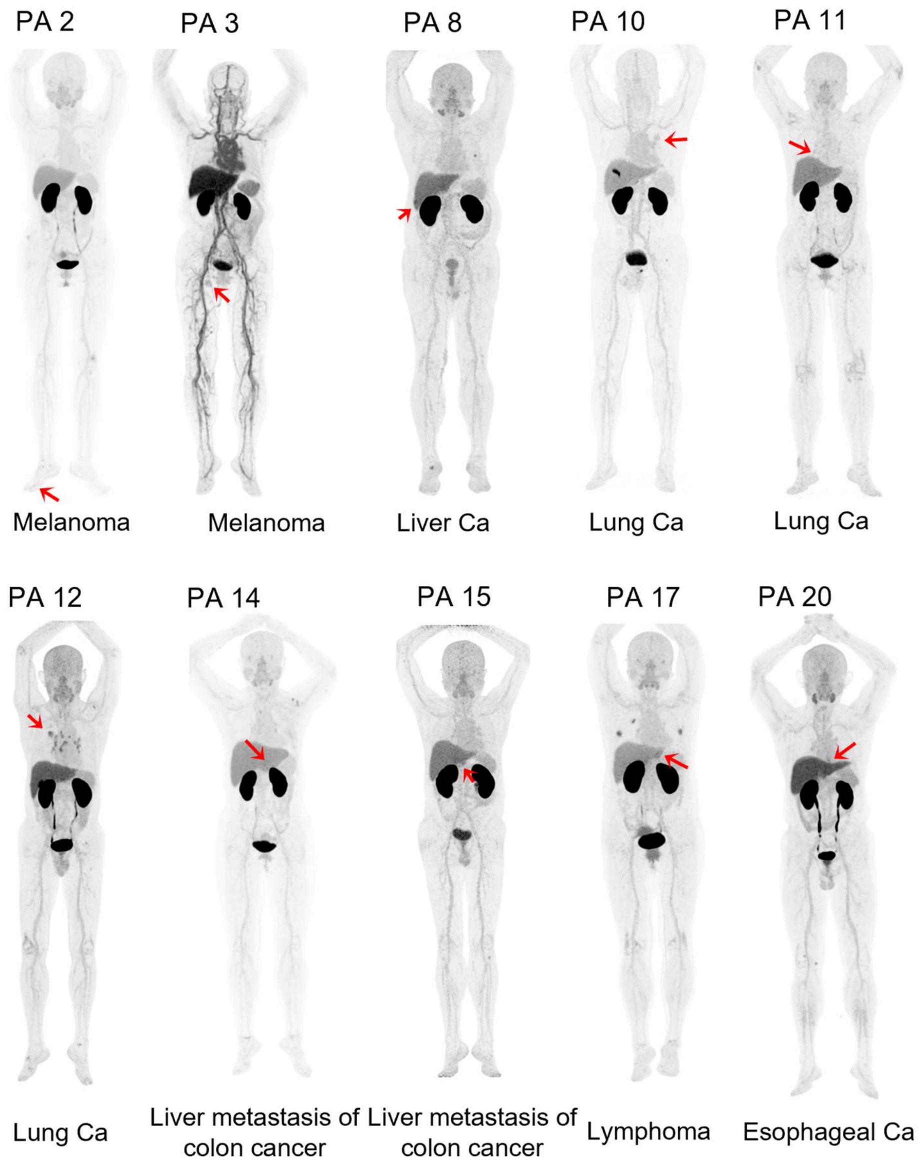

Fig. S15 Maximum intensity projection (MIP) images from **^{68}Ga-B7H3-BCH** PET scans of ten other patients, with red arrows highlighting both primary and metastatic lesions.

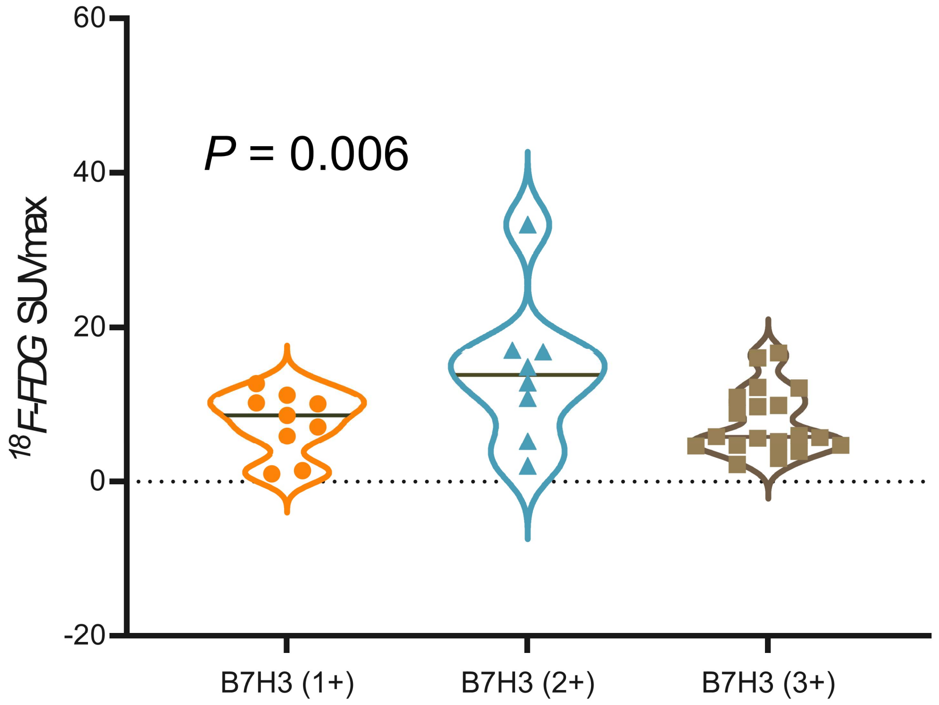

Fig. S16 Box plots depicting the SUVmax of ^{18}F-FDG for all 40 lesions in 12 patients with B7H3 *3+*, B7H3 *2+*, and B7H3 *1+* by IHC staining. Statistical significance was indicated using a hypothesis test on a linear mixed effect model ($P < 0.005$ was considered significant).

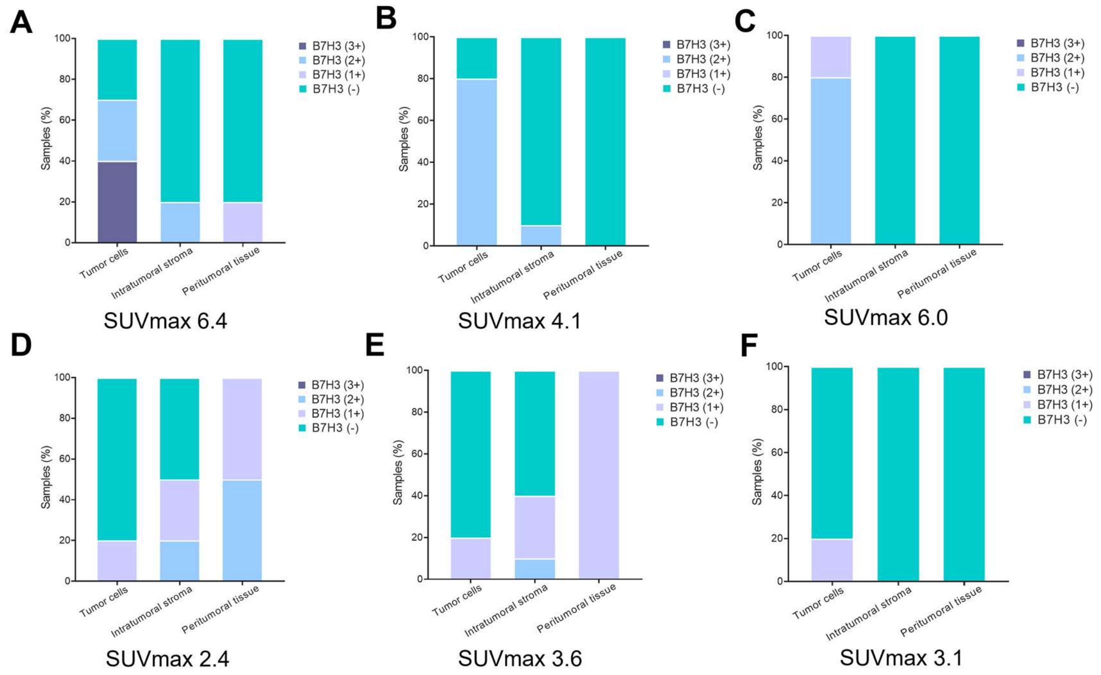

Fig. S17 Immunohistochemical analysis of tumor cells, intratumoral stroma, and peritumoral tissue with B7H3 expression graded as *3+*, *2+*, *1+*, and negative.

SUPPLEMENTARY TABLES

Table S1. Biodistribution of 68Ga-DOTA-AC12 in normal mice (%ID/g, Mean ± SD, n = 3)

Organ	5 min	30 min	60 min	120 min	240 min
**Blood**	10.36±1.48	4.28 ±0.64	1.82 ±0.08	1.95 ±1.22	0.70 ±0.16
**Heart**	8.39±1.31	3.30 ±0.27	2.42 ±0.33	1.92 ±0.36	0.82 ±0.55
**Liver**	6.81±0.79	2.75 ±0.21	1.97 ±0.52	2.72 ±0.37	1.38 ±0.25
**Spleen**	5.01±0.79	2.75 ±0.64	1.97 ±0.11	2.00 ±0.39	1.37 ±0.21
**Lung**	10.41 ±4.46	5.22 ±0.80	5.29 ±1.97	2.69 ±0.62	1.25 ±0.39
**Kidney**	267.92 ±30.18	496.68 ±89.13	633.90 ±6.28	721.66 ±17.94	441.75 ±89.81
**Stomach**	5.67 ±2.92	3.04 ±0.65	1.90 ±0.69	2.31 ±0.54	0.95 ±0.10
**SI**	11.62 ±1.18	7.51 ±3.22	4.09 ±0.40	3.54 ±0.49	1.73 ±0.03
**LI**	12.29 ±0.89	7.83 ±1.41	5.08 ±0.40	5.18 ±0.66	2.71 ±0.37
**Muscle**	10.51 ±0.74	4.67 ±0.84	8.52 ±0.80	4.35 ±1.17	5.59 ±2.26
**Bone**	18.98 ±2.37	20.33 ±0.38	21.22 ±2.33	9.68 ±1.10	11.86 ±2.77
**Brain**	0.68 ±0.10	0.47 ±0.03	0.76 ±0.58	0.61 ±0.28	0.36 ±0.03

Table S2. Biodistribution of 68Ga-DOTA-AC12 in normal mice (%ID/g, Mean ± SD, n = 3)

Organ	5 min	30 min	60 min	120 min	240 min
**Blood**	6.64±1.11	1.05±0.22	0.62±0.24	0.36±0.17	0.20±0.02
**Heart**	4.07±0.65	0.95±0.17	0.61±0.14	0.88±0.03	0.67±0.03
**Liver**	3.83±0.70	1.86±0.09	1.46±0.14	1.20±0.60	0.62±0.20
**Spleen**	3.47±0.21	1.65±0.35	1.24± 0.07	2.26±0.63	1.45±0.18
**Lung**	25.70±6.06	20.77±4.59	12.35 ±2.37	9.59±3.68	5.70±3.10
**Kidney**	146.63±2.35	160.76±10.41	104.02±19.57	63.48±8.89	26.55±6.88
**Stomach**	2.90±0.35	0.79±0.12	0.61 ±0.31	0.67±0.08	0.43±0.16
**SI**	5.31±0.46	2.01±0.49	1.90±0.78	3.38±1.28	1.49±0.22
**LI**	5.06±0.20	1.87±0.31	1.73±0.74	2.20±0.50	1.76±0.08
**Muscle**	3.08±0.19	1.57±0.26	2.23 ±0.32	3.26±0.84	2.64±0.92
**Bone**	6.07±1.36	4.21±0.40	8.05±2.93	11.50±0.71	8.62±2.29
**Brain**	0.52±0.18	0.15±0.00	0.31±0.14	0.49±0.07	0.38±0.16

Table S3. Quality control of ^{68}Ga-B7H3-BCH used in this study.

Parameter	QC Specification	QC Result
**Appearance**	Clear, colorless	Pass
**Volume**	2.0-10.0 mL	4.5 mL
**pH**	5.0-8.0	7.4
**Radio-TLC**	>95%	>99%
**Radio-HPLC**	>95%	>99%
**Ethanol**	<10%	8%
**Specific Activity**	40-120 GBq/μmol	53.3 GBq/μmol

Table S4. Estimation of radiation dosimetry in human organs

Target Organ	mGy/MBq
**Adrenals**	1.06E-01
**Brain**	1.15E-02
**Breasts**	1.09E-02
**Esophagus**	1.51E-02
**Eyes**	1.06E-02
**Gallbladder Wall**	4.13E-02
**Left colon**	2.26E-02
**Small Intestine**	2.58E-02
**Stomach Wall**	2.15E-02
**Right colon**	2.23E-02
**Rectum**	1.76E-02
**Heart Wall**	1.55E-02
**Kidneys**	1.87E+00
**Liver**	3.78E-02
**Lungs**	6.93E-03
**Ovaries**	1.56E-02
**Pancreas**	2.95E-02
**Salivary Glands**	1.10E-02
**Red Marrow**	1.46E-02
**Osteogenic Cells**	2.01E-02
**Spleen**	5.83E-02
**Thymus**	1.17E-02
**Thyroid**	1.13E-02
**Urinary Bladder Wall**	1.22E-02
**Uterus**	1.49E-02
**Total Body**	2.50E-02
**Effective Dose**	3.31E-02

Table S5. Estimation of radiation dosimetry in human organs

Target Organ	mGy/MBq
**Adrenals**	3.15E-02
**Brain**	7.48E-03
**Breasts**	8.10E-04
**Esophagus**	3.02E-03
**Eyes**	6.72E-04
**Gallbladder Wall**	1.02E-02
**Left colon**	3.79E-03
**Small Intestine**	6.46E-03
**Stomach Wall**	4.32E-03
**Right colon**	3.72E-03
**Rectum**	2.90E-03
**Heart Wall**	5.25E-03
**Kidneys**	5.94E-01
**Liver**	1.81E-02
**Lungs**	2.61E-02
**Ovaries**	1.31E-03
**Pancreas**	6.51E-03
**Salivary Glands**	6.12E-04
**Red Marrow**	2.61E-03
**Osteogenic Cells**	8.03E-03
**Spleen**	1.95E-02
**Thymus**	2.63E-03
**Thyroid**	1.12E-03
**Urinary Bladder Wall**	5.83E-04
**Uterus**	1.12E-03
**Total Body**	5.89E-03
**Effective Dose**	1.19E-02

Table S6. Radiation dosimetry in human organs (Female=1, Male =2)

Target Organ	mGy/MBq
**Adrenals**	4.25E-01
**Brain**	2.45E-04
**Breasts**	2.95E-03
**Esophagus**	1.26E-02
**Eyes**	2.50E-04
**Gallbladder Wall**	5.10E-02
**Left colon**	3.83E-02
**Small Intestine**	2.71E-02
**Stomach Wall**	2.44E-02
**Right colon**	3.03E-02
**Rectum**	9.23E-03
**Heart Wall**	1.19E-02
**Kidneys**	5.78E+00
**Liver**	4.74E-02
**Lungs**	7.74E-03
**Ovaries**	2.81E-02
**Pancreas**	3.99E-02
**Prostate**	2.94E-02
**Salivary Glands**	5.70E-04
**Red Marrow**	1.47E-02
**Osteogenic Cells**	9.99E-03
**Spleen**	1.25E-01
**Testes**	4.70E-03
**Thymus**	4.63E-03
**Thyroid**	5.88E-03
**Urinary Bladder Wall**	9.84E-03
**Total Body**	3.49E-02
**Effective Dose**	7.02E-02

Table S7. Information of patients with malignant tumors enrolled in PET/CT imaging studies using ^{68}Ga-B7H3-BCH and ^{18}F-FDG

	Gender	Age(year)	Weight (kg)	Dose (Mbq)	^{68}Ga-B7H3-BCH protocal	^{18}F-FDG protocal	Pathological
**1**	Male	66	68	95.6	Static (50-60min)	Static(50-60min)	**Melanoma**
**2**	Female	59	53	74.1	Static (50-60min)	Static(50-60min)	**Melanoma**
**3**	Female	75	65	92.1	Static (50-60min); Static (120-125min)	Static(50-60min)	**Melanoma**

**4**	Female	52	63	87.6	Static (50-60min); Static (120-125min)	Static(50-60min)	**Adenocarcinoma**
**5**	Female	60	62	87.2	Static (50-60min)	Static(50-60min)	**Stomach cancer**
**6**	Male	60	65	91.2	Static (50-60min)	Static(50-60min)	**esophageal cancer**
**7**	Female	60	65	94.3	Dynamic (0-50min); Static (120-125min)	Static(50-60min)	**Liver cancer**
**8**	Male	56	104	138.9	Static (50-60min); Static (120-125min)	Static(50-60min)	**Liver cancer**
**9**	Male	66	57	80.9	Static (50-60min)	Static(50-60min)	**Lung cancer**
**10**	Male	64	80	115.2	Static (50-60min); Static (120-125min)	Static(50-60min)	**Lung cancer**
**11**	Female	61	69	94.8	Static (50-60min); Static (120-125min)	Static(50-60min)	**Lung cancer**
**12**	Male	65	65	92.6	Static (50-60min)	Static(50-60min)	**Lung cancer**
**13**	Male	64	90	126	Dynamic (0-50min); Static (120-125min)	Static(50-60min)	**Stomach cancer and Colon cancer**
**14**	Male	72	48	75.3	Static (50-60min)	Static(50-60min)	**Colon cancer**
**15**	Male	36	60	83.2	Static (50-60min)	Static(50-60min)	**Colon cancer**
**16**	Female	70	60	87.1	Dynamic (0-50min); Static (120-125min)	Static(50-60min)	**lymphoma**
**17**	Female	50	72	100.4	Static (50-60min)	Static(50-60min)	**lymphoma**
**18**	Female	60	63	88.1	Static (50-60min)	Static(50-60min)	**Breast cancer**
**19**	Male	70	75	104.2	Static (50-60min)	Static(50-60min)	**rectal cancer**
**20**	Male	66	70	98.2	Static (50-60min)	Static(50-60min)	**esophageal cancer**

Table S8. Comparison of primaries and metastases on [^{68}Ga]Ga-B7H3-BCH and ^{18}F-FDG PET/CT via Lesion-Based visual analysis

Regions	B7H3+/FDG+	B7H3+/FDG-	B7H3-/FDG+
**Primary**	17	1	2
**Metastase**	25	8	5
**Total**	42	9	7
