# Supplementary material for: A whole-body imaging technique for tumor-specific diagnostics and screening of B7H3-targeted therapies
Source: J Clin Invest. 2025 Jan 23;135(6):e186388. doi: 10.1172/JCI186388 (PMC11910224; doi:10.1172/JCI186388)
Supplement: Unedited blot and gel images [file jci-135-186388-s239.pdf]

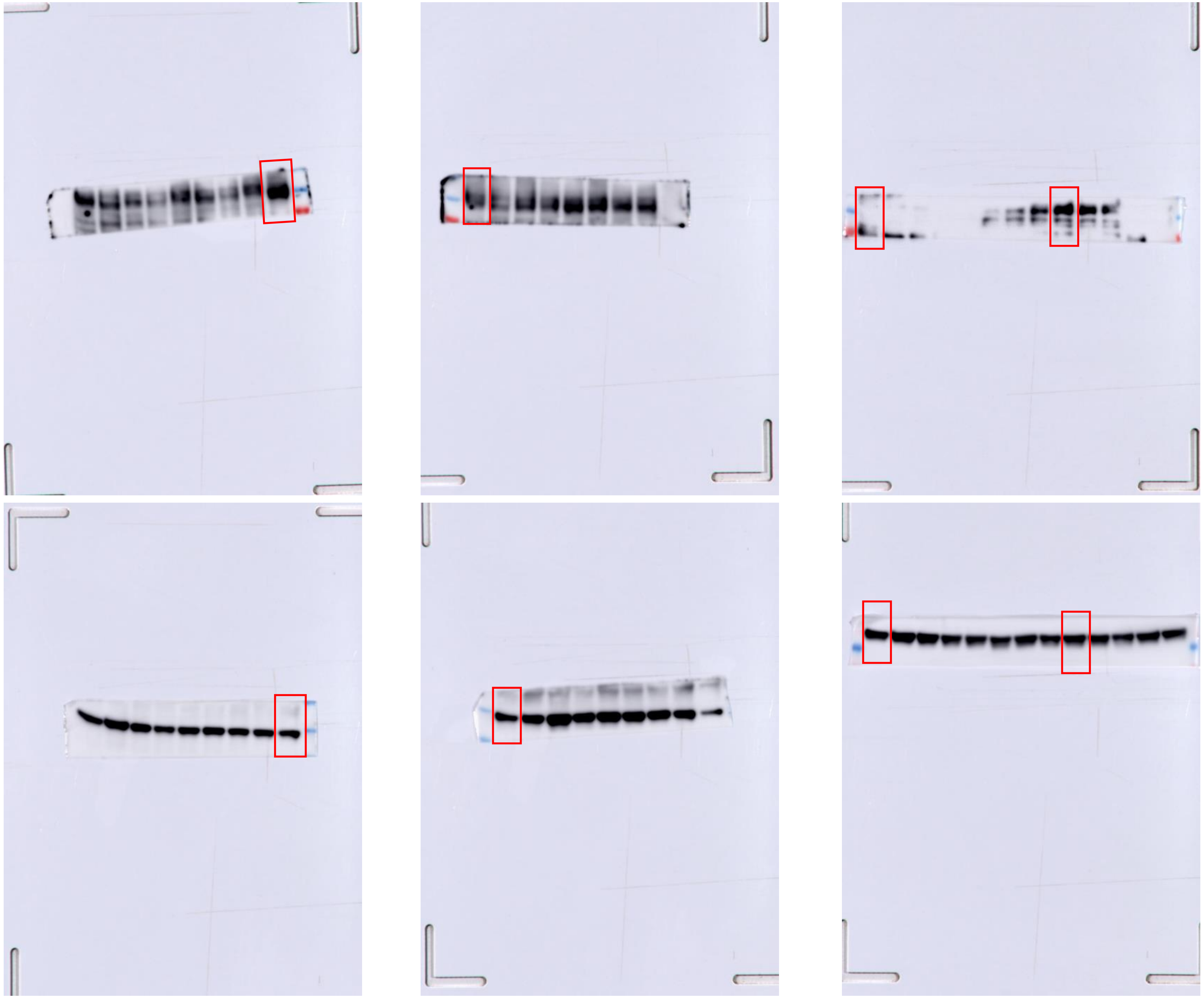

The full unedited gel for Figure 2B

The target band is within the red box, with the experimental antibody: 14058, CST, USA.
